# Sex and age differences in the Multiple Sclerosis prodrome

**DOI:** 10.3389/fneur.2022.1017492

**Published:** 2022-11-03

**Authors:** Fardowsa L. A. Yusuf, José M. A. Wijnands, Mohammad Ehsanul Karim, Elaine Kingwell, Feng Zhu, Charity Evans, John D. Fisk, Yinshan Zhao, Ruth Ann Marrie, Helen Tremlett

**Affiliations:** ^1^Division of Neurology, Department of Medicine, The Djavad Mowafaghian Centre for Brain Health, University of British Columbia, Vancouver, BC, Canada; ^2^School of Population and Public Health, University of British Columbia, Vancouver, BC, Canada; ^3^Centre for Health Evaluation and Outcome Sciences, St. Paul's Hospital, Vancouver, BC, Canada; ^4^Research Department of Primary Care & Population Health, University College London, London, United Kingdom; ^5^College of Pharmacy and Nutrition, University of Saskatchewan, Saskatoon, SK, Canada; ^6^Nova Scotia Health and the Department of Psychiatry, Psychology & Neuroscience, and Medicine, Dalhousie University, Halifax, NS, Canada; ^7^Department of Internal Medicine and Community Health Sciences, Rady Faculty of Health Sciences, Max Rady College of Medicine, University of Manitoba, Health Sciences Centre, Winnipeg, MB, Canada

**Keywords:** Multiple Sclerosis, prodromal, age, sex, healthcare use

## Abstract

**Background and objectives:**

Little is known of the potential sex and age differences in the MS prodrome. We investigated sex and age differences in healthcare utilization during the MS prodrome.

**Methods:**

This was a population-based matched cohort study linking administrative and clinical data from British Columbia, Canada (population = 5 million). MS cases in the 5 years preceding a first demyelinating event (“administrative cohort;” *n* = 6,863) or MS symptom onset (“clinical cohort;” *n* = 966) were compared to age-, sex- and geographically-matched controls (*n* = 31,865/4,534). Negative binomial and modified Poisson models were used to compare the rates of physician visits and hospitalizations per international classification of diseases chapter, and prescriptions filled per drug class, between MS cases and controls across sex and age-groups (< 30, 30–49, ≥50 years).

**Results:**

In the administrative cohort, males with MS had a higher relative rate for genitourinary-related visits (males: adjusted Rate Ratio (aRR) = 1.65, females: aRR = 1.19, likelihood ratio test *P* = 0.02) and antivertigo prescriptions (males: aRR = 4.72, females: aRR = 3.01 *P* < 0.01). Injury and infection-related hospitalizations were relatively more frequent for ≥50-year-olds (injuries < 30/30–49/≥50: aRR = 1.16/1.39/2.12, *P* < 0.01; infections 30–49/≥50: aRR = 1.43/2.72, *P* = 0.03), while sensory-related visits and cardiovascular prescriptions were relatively more common in younger persons (sensory 30–49/≥50: aRR = 1.67/1.45, *P* = 0.03; cardiovascular < 30/30–49/≥50: aRR = 1.56/1.39/1.18, *P* < 0.01). General practitioner visits were relatively more frequent in males (males: aRR = 1.63, females: aRR = 1.40, *P* < 0.01) and ≥50-year-olds (< 30/≥50: aRR = 1.32/1.55, *P* = 0.02), while differences in ophthalmologist visits were disproportionally larger among younger persons, < 50-years-old (< 30/30–49/≥50: aRR = 2.25/2.20/1.55, *P* < 0.01). None of the sex and age-related differences in the smaller clinical cohort reached significance (*P* ≥ 0.05).

**Discussion:**

Sex and age-specific differences in healthcare use were observed in the 5 years before MS onset. Findings demonstrate fundamental heterogeneity in the MS prodromal presentation.

## Introduction

Multiple Sclerosis (MS) is a chronic, immune-mediated central nervous system disorder. In recent years, evidence has emerged of a measurable, symptomatic phase of MS that precedes the classic understanding of MS disease onset, a period known as a prodrome (1). Several studies have found increased total and condition-specific health-care utilization in the years preceding MS onset and diagnosis (1–6). Biological changes are also evident during this period, including higher serum neurofilament light levels up to 6 years before MS symptom onset (vs. matched persons without MS) (7). Distinct peripheral T memory cell signatures were also observed in monozygotic twin pairs discordant for MS, where the clinically healthy cotwin had subclinical signs of prodromal MS (8). In addition, predictive performance of these health-care data in discriminating between people with MS and the general population holds promise (9, 10).

Other neurological conditions, such as Parkinson's disease, have well-defined prodromal phases, the characteristics of which differ by sex and age (11, 12). Given that sex and age are associated with phenotypic and MRI-related structural differences even at MS symptom onset (13, 14), it is likely that such differences predate onset and are present in the prodromal phase. Further, a recent study has shown that younger age and male sex are prognostic factors for the earlier clinical evolution from radiologically isolated syndrome to MS (15), suggesting that the intermediary prodromal phase is experienced differently for males and females and across age groups. To date, few studies have examined sex and age-related heterogeneity in the MS prodrome. Studies that have done so have focused on a limited set of conditions (5). We aimed to examine the potential for sex and age-specific differences of the MS prodrome across a wide range of features by using comprehensive population-based linked health service use information and MS-specific clinical data from British Columbia, Canada.

## Materials and methods

### Study design and data sources

Using a matched cohort design, we assessed sex- and age-related differences in health-care utilization between individuals with MS and matched general population controls in the 5 years before the first demyelinating diagnostic code or MS symptom onset. Previous studies have found increased health-utilization for various diseases over the same 5-year study period (2, 3).

Linked health administrative data from the Canadian province of British Columbia (BC) were accessed. These comprised physician visits (16), hospitalizations (17), prescriptions filled (18), and (19) dates of registration in the compulsory, universal health system. Dates of physician encounters and hospital admissions, with the relevant International Classification of Diseases (ICD)-9/10 diagnostic codes were accessed, as were drug identification numbers and dates of prescriptions filled in community and out-patient pharmacies. In addition, we accessed each participant's sex, date of birth, place of residence (postal code) and neighborhood income quintile [as an indicator of socioeconomic status (SES)]. The total days registered in the health system each year was accessed for evidence of residency in the province. Data were available from 1-April-1991 until 31-December-2013, except for prescriptions (available from 1-January-1996).

The health administrative data were also linked to a cohort of individuals who had visited a MS clinic in BC from 1-January-1991 to 31-December-2008. MS-specific clinical data, including MS symptom onset date as recorded by the patient's MS neurologist, were available and accessed for this cohort. This study was approved by the University of British Columbia Clinical Research Ethics Board and British Columbia Ministry of Health. Written informed patient consent was obtained in accordance with the requirements of the institutional ethics board.

### Identifying cases and controls

A population-based health administrative cohort and a smaller clinical MS cohort were identified, as detailed previously (2, 3, 5). Briefly, for the administrative cohort, MS cases were required to fulfill a validated algorithm requiring ≥3 MS-specific hospital, physician or prescription records (Supplementary Table S1) (20). For the clinical cohort, MS cases were required to have visited an MS clinic in BC and to have received an MS diagnosis from an MS specialist neurologist. Each MS case was assigned an index date; for the health administrative cohort, this was the date of the first demyelinating disease-related diagnostic code (“event”), Supplementary Table S1, and for the clinical cohort, this was the date of MS symptom onset.

Up to 5 randomly-selected general population controls in BC were matched to each MS case by sex, birth year and the first 3 characters of the postal code at the index year. Controls were required to have had no demyelinating disease-related physician or hospital diagnostic codes and to have never filled an MS-specific drug prescription (Supplementary Table S1). Each control was assigned the index date of their respective case. Both the cases and controls had to be resident in BC for ≥90% of the days in each of the 5 years preceding the index date.

### Outcomes

The study outcomes included the total number of physician visits and hospital admissions (which also served as measures of the overall healthcare burden), and the “reason” for the healthcare use, measured as the number per ICD-9/10 chapter, in the 5-years pre-index-date (Supplementary Tables S2, S3). Physician visits per specialty (Supplementary Table S3) were also examined, as were prescriptions as “any” dispensation (“fill”), and fills by drug class [Anatomical Therapeutic Chemical (ATC) level 1 (21), Supplementary Table S4], categorized as “yes/no.”

### Statistical analyses

The characteristics, at the index date, of individuals in the two cohorts were summarized. In the 5 years before the administrative or clinical index dates, the rates of each study outcome were compared between the MS cases and controls by sex (males, females) and across age groups at the index date (categorized as < 30, 30–49, ≥50 years). This was performed by introducing interaction terms between sex and MS disease status, and age and MS disease status. We did not consider further interactions between age, sex and MS, given the large number of possible outcomes. Sex- and age-specific adjusted rate ratios (aRRs) and 95% confidence intervals (CI) were obtained from negative binomial regression models for physician and hospital encounters and from Poisson regression models with robust error variances (22) for prescriptions filled. Models also accounted for the variation in the time spent resident in the province and the number of controls per case. We controlled for confounding by sex, as we calculated relative rates comparing males with MS to male controls, and females with MS to female controls.

Covariates included sex, and at the index date, age, SES quintiles (reference category = lowest quintile) and year (grouped as 1996–2000, 2001–2005, 2006–2008/2010, 2011–2013; reference category = 1996–2000). Sex-specific models were adjusted for age, and age-specific models were adjusted for sex. Models with and without the sex- or age-specific interaction term were compared using likelihood ratio tests with a significance level of 0.05. *Post-hoc* Bonferroni adjusted analyses were applied to determine which age-groups differed.

In complementary analyses, ICD-9 sub-chapters for physician encounters, ATC level 3 prescription classes (21) and the prevalence of 14 morbidities identified *via* validated health administrative algorithms (Supplementary Tables S5–S7), were examined in the 5-years pre-index date in the larger health administrative cohort only.

Analyses were conducted in R version 4.0.3.

## Results

The administrative cohort included 6,863 MS cases and 31,865 matched controls, and the clinical cohort included 966 MS cases and 4,534 matched controls (Table 1). As expected, the clinical cohort was younger than the administrative cohort at their respective index dates (mean age at MS symptom onset = 37 years and at a first demyelinating event = 44 years). Females comprised over 73% of both cohorts. The distribution of SES quintiles was similar between cohorts.

**Table 1:** Characteristics of the multiple sclerosis (MS) cases and controls in the administrative and clinical cohorts at the index date in British Columbia, Canada.

	**Administrative cohort**		**Clinical cohort**	
**Characteristics**	**MS cases,** ***N* = 6,863**	**Controls,** ***N* = 31,865^**c**^**	**MS cases,** ***N* = 966**	**Controls,** ***N* = 4,534^**c**^**
Females, *N* (%)	5,039 (73.4)	23,311 (73.2)	728 (75.4)	3,398 (74.9)
Age at index date (years)				
< 30	859 (12.5)	4,080 (12.8)	232 (24.0)	1,082 (23.9)
30 to < 50	3,827 (55.8)	17,899 (56.2)	639 (66.1)	2,992 (66.0)
≥50	2,177 (31.7)	9,886 (31.0)	95 (9.8)	460 (10.1)
Mean (SD)	44.4 (13.5)	44.2 (13.4)	37.0 (10.2)	36.9 (10.1)
Index year, *N* (%)				
1996–2000	2,001 (29.2)	9,216 (28.9)	589 (61.0)	2,764 (61.0)
2001–2005	2,149 (31.3)	9,914 (31.1)	344 (35.6)	1,618 (35.7)
2006–2008^a^/2010^b^	2,078 (30.3)	9,736 (30.6)	33 (3.42)	152 (3.35)
2011–2013	235 (9.3)	2,999 (9.4)		
SES at index date, *N* (%)				
1 (lowest quintile)	1,184 (17.3)	5,476 (17.2)	145 (15.0)	763 (16.8)
2	1,286 (18.7)	5,950 (18.7)	180 (18.6)	812 (17.9)
3	1,413 (20.6)	6,329 (19.9)	204 (21.1)	895 (19.7)
4	1,465 (21.3)	6,826 (21.4)	194 (20.1)	962 (21.2)
5 (highest quintile)	1,365 (19.9)	6,591 (20.7)	210 (21.7)	952 (21.0)
Missing	150 (2.2)	693 (2.2)	33 (3.4)	150 (3.3)

^*a*^The index year category for the clinical cohort was 2006–2008.

^*b*^The index year category for the administrative cohort was 2006–2010.

^*c*^Five controls matching on sex, birth year and postal code were not available for each MS case. Therefore, some MS cases were matched to fewer than 5 controls.

SES, socio-economic status.

### Administrative cohort

#### Sex-related differences

In the 5-years preceding a first demyelinating event, relative rates comparing males with MS to males in the general population were 15% greater for total physician visits, and 21% higher for total hospital admissions, than relative rates for females (Table 2). Males also had a 4% greater relative rate for filling a prescription than females.

**Table 2:** Sex and age-specific differences in health-care use for any reason between multiple sclerosis cases and controls: 5 years before the index date in the health administrative and clinical cohorts.

		**Administrative cohort**			**Clinical cohort**		
		**MS cases^**a**^**	**Controls^**a**^**		**MS cases^**a**^**	**Controls^**a**^**	
		**Crude rate** **(per person-year)/** **crude proportion (%)**	**Crude rate** **(per person-year)/** **crude proportion (%)**	**aRR (95% CI)**	**Crude rate** **(per person-year)/** **crude proportion (%)**	**Crude rate** **(per person-year)/** **crude proportion (%)**	**aRR (95% CI)**
Physician visits	Males	11.4	6.83	**1.67 (1.57–1.77)** ^**b**^	6.70	6.19	1.09 (0.91–1.30)
	Females	14.9	10.2	**1.45 (1.40–1.51)** ^**b**^	12.4	10.5	**1.17 (1.06–1.30)**
	< 30	10.5	7.72	**1.39 (1.27–1.52)** ^**b**^	9.34	8.41	1.13 (0.94–1.35)
	30–49	13.1	8.87	**1.49 (1.43–1.55)** ^**b**^	11.1	9.69	**1.14 (1.02–1.27)**
	≥50	16.9	10.7	**1.58 (1.49–1.68)** ^**b**^	13.9	10.4	1.28 (0.97–1.68)
Hospital admissions	Males	0.202	0.117	**1.73 (1.54–1.95)** ^**b**^	0.0832	0.0896	0.93 (0.61–1.45)
	Females	0.249	0.171	**1.43 (1.34–1.54)** ^**b**^	0.194	0.181	1.07 (0.88–1.30)
	< 30	0.173	0.130	**1.35 (1.13–1.62)** ^**b**^	0.144	0.143	1.02 (0.70–1.48)
	30–49	0.207	0.145	**1.43 (1.32–1.55)** ^**b**^	0.161	0.161	1.00 (0.80–1.23)
	≥50	0.312	0.189	**1.65 (1.49–1.82)** ^**b**^	0.261	0.174	1.40 (0.84–2.34)
Any prescription filled	Males	94.0	87.3	**1.08 (1.06–1.09)** ^**b**^	82.8	80.9	1.02 (0.96–1.09)
	Females	97.2	93.0	**1.04 (1.04–1.05)** ^**b**^	93.8	89.9	**1.04 (1.02–1.07)**
	< 30	96.7	91.9	**1.05 (1.04–1.07)**	91.8	89.6	1.02 (0.98–1.07)
	30–49	95.6	90.5	**1.06 (1.05–1.06)**	90.8	86.3	**1.05 (1.02–1.08)**
	≥50	97.6	93.1	**1.05 (1.04–1.06)**	91.6	91.7	1.00 (0.93–1.07)

aRR, adjusted rate ratio; CI, confidence interval.

bold = P < 0.05 for sub-group.

^*a*^physician visits and hospital admissions represented as crude rates; “any” prescription filled represented as crude proportions.

^*b*^Based on the likelihood ratio test, the rate ratios statistically differed (P < 0.05) between males and females or between the age categories: < 30, 30–49 and ≥50 years.

The post-hoc Bonferroni adjusted P-values derived when each age-group was directly compared against each other in the administrative cohort were as follows: physician visits (< 30 vs. 30–49: P = 0.54; < 30 vs. ≥50: P = 0.05; 30-49 vs. ≥50: P = 0.27), hospitalizations (< 30 vs. 30-49: P = 1; < 30 vs. ≥50: P = 0.14; 30-49 vs. ≥50: P = 0.10), prescriptions-filled (< 30 vs. 30–49: P = 1.00; < 30 vs. ≥50: P = 1.00; 30–49 vs. ≥50: P = 0.58).

**Figure 1:**
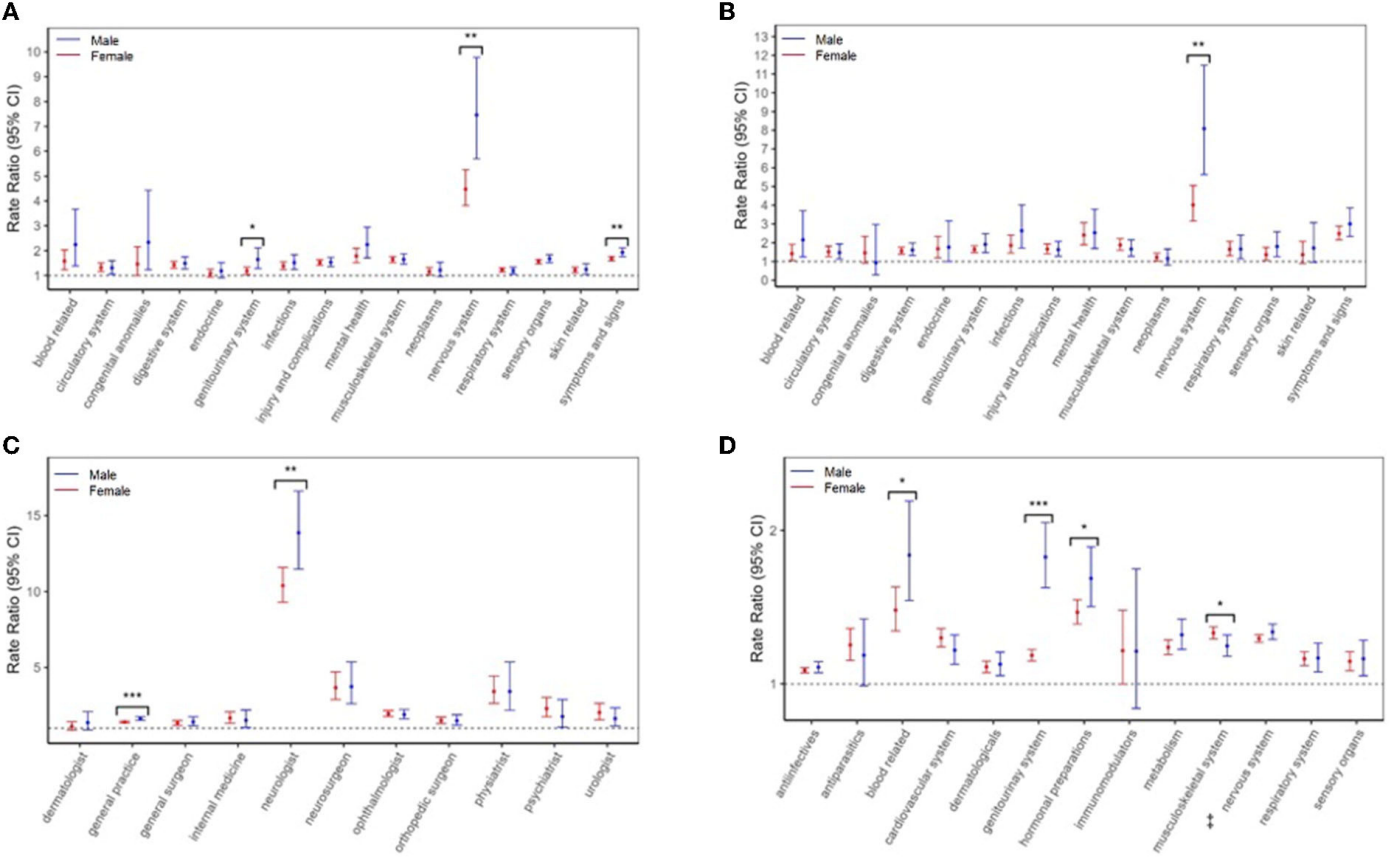
Multiple sclerosis (MS) cases vs. controls in the 5 years before the first demyelinating diagnostic code: sex-specific rate ratios for **(A)** physician visits and **(B)** hospitalizations per International Classification of Diseases-9/10 chapter, **(C)** physician visits per specialty, and **(D)** prescriptions-filled per Anatomical Therapeutic Chemical (ATC) level 1 classification. Results were not reported if a model failed to converge due to low counts or the 95% CIs were wide (exceeded 100). *P < 0.05, **P < 0.01, *** *P* < 0.001 indicates that the rate ratios were statistically different between men and women, based likelihood ratio tests. ^‡^Amantadine was categorized as a nervous system drug, based on the Anatomical Therapeutic Chemical (ATC) level I classification system.

Sex differences were observed across all health-care sectors when the reasons for that health-care use were examined (*P* < 0.05; Figure 1). In particular, males had 1.7 to 2 times the relative rate vs. females for nervous system-related hospitalizations and physician visits (males: aRR = 8.08 and 7.46, females: aRR = 4.01 and 4.48, respectively). Moreover, males had higher relative rates for genitourinary-related visits (males: aRR = 1.65, females: aRR = 1.19) and “other” ill-defined symptoms/signs (males: aRR = 1.93, females: aRR = 1.68). Higher relative rates for visits to general practitioners (GP) (males: aRR = 1.63, females: aRR = 1.40) and neurologists (males: aRR = 13.9, females: aRR = 10.4) were also found for males. The relative difference in the rates of filling a genitourinary drug-related prescription (males: aRR = 1.83; females: aRR = 1.18), hormonal preparation (males: aRR = 1.69; females: aRR = 1.47) or a blood-related agent (males: aRR = 1.84; females: aRR =1 .48) was significantly higher among males. In contrast, females had a higher relative rate for filling a musculoskeletal-related prescription (males: aRR = 1.25, females: aRR = 1.33).

#### Age-related differences

In the 5-years before a first demyelinating event, the relative difference between MS cases and controls in the total number of physician visits and hospitalizations differed across age groups based on the likelihood ratio test. However, these differences were not statistically significant following *post-hoc* Bonferroni-adjusted pairwise analyses. There were no age-based differences for the prescriptions filled.

**Figure 2:**
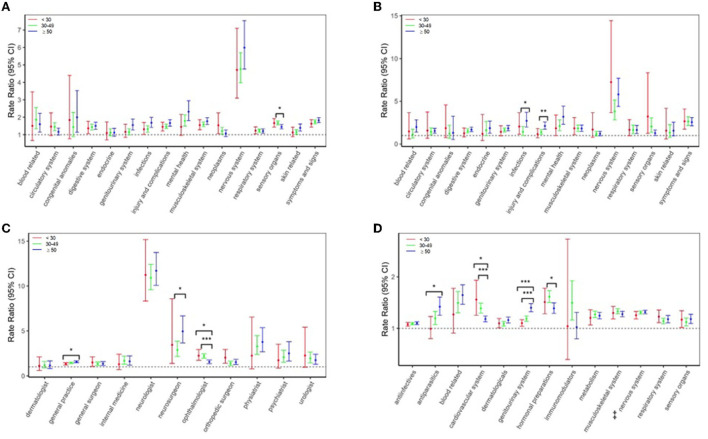
Multiple Sclerosis (MS) cases vs. controls in the 5 years before the first demyelinating diagnostic code: age-specific rate ratios for **(A)** physician visits and **(B)** hospitalizations per International Classification of Diseases-9/10 chapter, **(C)** physician visits per specialty, and **(D)** prescriptions-filled per Anatomical Therapeutic Chemical (ATC) level 1 classification. Results were not reported if a model failed to converge due to low counts or the 95% CIs were wide (exceeded 100). *P < 0.05, **P < 0.01, *** *P* < 0.001 indicates that the rate ratios are statistically different across age groups, based on Bonferroni tests following likelihood ratio tests. ^**‡**^Amanradine was categorized as a nervous system drug, based on the Anatomical Therapeutic Chemical (ATC) level 1 classification system.

When the specific reasons for health-care use were examined (Figure 2), relative rates for hospitalizations due to injuries/complications were higher for individuals aged ≥50 years (aRR = 2.12) than the younger age groups (< 30 years: aRR = 1.16, 30–49 years: aRR = 1.39), and infections were higher (aRR = 2.72) than the 30–49-year-olds (aRR = 1.43). Relative rates for, e.g., GP visits were also higher for ≥50 year-olds (aRR = 1.55) than < 30-year-olds (aRR = 1.32), as were some prescription fills, e.g., genitourinary drugs (aRR = 1.40) than either the < 30-year-olds (aRR = 1.10) or 30–49-year-olds (aRR = 1.19).

However, relative rates for ophthalmologist visits were higher in both younger age groups (< 30 years: aRR = 2.25, 30–49 years: aRR = 2.20) vs. the ≥50-year-olds (aRR = 1.55) and higher for sensory organ-related physician visits (aRR = 1.67) than ≥50-year-olds (aRR = 1.45). The diagnostic codes recorded by ophthalmologists included those for retinal and corneal disorders, but not uveitis. In addition, the relative rate for several drugs, such as a cardiovascular-related prescription fill was also higher among younger persons (< 30 years: aRR = 1.56, 30–49 years: aRR = 1.39) than older (≥50 years: aRR = 1.18).

### Clinical cohort

In the smaller clinical cohort, the rates for health-care utilization were significantly higher among MS cases than controls in some specific sex and age groups (Table 2, Supplementary Figures S1, S2). However, these relative rates did not differ statistically between males and females or by age. For example, although females with MS had significantly higher relative rates for musculoskeletal-related physician visits (aRR = 1.30), and hormonal-related prescriptions (aRR = 1.31) than females without MS, the same was not observed in males, and the relative rates did not differ between sexes (*P* ≥ 0.05). Similarly, while urologist visits were higher across all age groups with MS (vs. controls), reaching statistical significance for the 30–49-year-olds (aRR = 1.90), relative rates were not statistically different between age groups. Nonetheless, similar patterns between the clinical and administrative cohorts were observed for the total number of physician visits and hospitalizations, as well as cardiovascular and blood related prescription fills, albeit all *P* ≥ 0.05.

### Complementary analyses

In the 5-years preceding a first demyelinating code, the relative difference in physician visits, assessed as ICD sub-chapter, between MS cases and controls was significantly larger among females (aRR = 1.65) than males (aRR = 1.11) for arthropathy-related visits only. Whereas, males had significantly higher relative rates than females for several conditions, including ill-defined/unknown causes of morbidity (males: aRR = 7.39; females: aRR = 1.58), peripheral nervous system disorders (males: aRR = 7.39; females: aRR = 4.86) and burns (males: aRR = 3.10; females: aRR = 1.39). Further, peripheral nervous system-related visits were more evident in < 30-year-olds (aRR = 9.78) vs. 30–49-year-olds (aRR = 5.58), as were burns (< 30 years: aRR = 4.39, 30–49 years: aRR = 1.19). Conditions more pronounced among ≥50-year-olds included skin-related disorders (aRR = 1.43), as compared to 30–49-year-olds (aRR = 1.11), and fractures of the spine/trunk (4.01) vs. both younger age groups (< 30 years: aRR = 0.52, 30–49 years: aRR = 1.13). See Supplementary Figure S3 for all ICD-9 sub-chapters which differed significantly by sex or age.

Prescriptions filled, by drug classes (ATC level 3), also differed by sex and age before a first demyelinating code. For example, females had higher relative rates for direct-acting antiviral drugs (males: aRR = 1.07; females: aRR = 1.43) and psychostimulants (males: aRR = 1.49; females: aRR = 2.92). While males had higher relative rates for antiepileptics (males: aRR = 3.44; females: aRR = 2.81), antidepressants (males: aRR = 1.90; females: aRR = 1.67) and antivertigo preparations (males: aRR = 4.72; females: aRR = 3.01). Moreover, < 30-year-olds had higher relative rates for antivertigo preparations (aRR = 6.66) than both older age groups (30–49 years: aRR = 3.44, ≥50 years: aRR = 2.64), but lower for antidepressants (aRR = 1.41) than ≥50-year-olds (aRR = 1.83). The relative rates for laxatives were greater among ≥50-year-olds (aRR = 2.28) than 30–49-year-olds (aRR = 1.30). See Supplementary Figure S4 for all drug classes which differed significantly by sex or age.

For the specific morbidities, males had two times the relative prevalence of migraine [males: adjusted Prevalence Ratio (aPR):3.72; females: aPR 1.84] and 1.21 times the relative prevalence of mood/anxiety disorders (males: aPR 2.36; females: aPR 1.95) preceding a first demyelinating code. The relative prevalence of mood/anxiety was higher for older persons (< 30 years: aPR 1.62; ≥50 years: aPR 2.18). Finally, the relative prevalence of ischemic heart disease (IHD) for females (aPR 1.49) was 38% greater than that for males (aPR 1.08) and 85% higher for 30–49-year-olds (aPR 2.11) than ≥50-year-olds (aPR 1.14). See Supplementary Figure S5.

**Figure 3:**
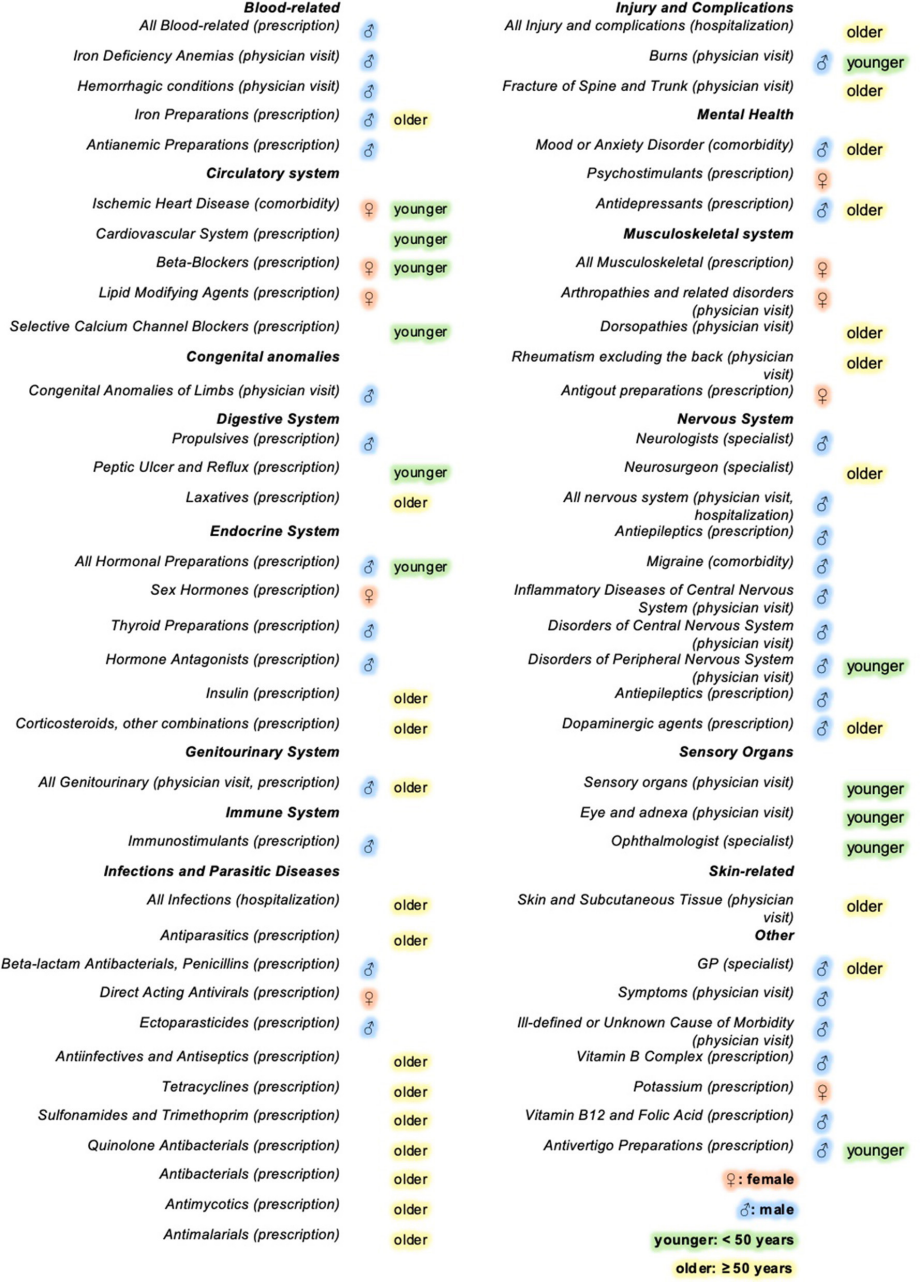
Infographic summarizing the sex and age differences in health-care use in the 5 years preceding a first demyelinating event, grouped by the main condition or body system affected^†‡^. The infographic depicts all the significant findings for the hospitalizations, physician visits, prescriptions filled and the comorbidities examined. For example, the relative rates of GP visits (shaded in blue), was greater in men with MS in the 5 years preceding a 1st demyelinating event than women with MS. ^†^Comorbidities are conditions identified in the 5-years preceding a 1st demyelinating event via validated health administrative algorithms. ^‡^Disease and drug classes, specialty visits and comorbidities were compared between MS cases and controls across sex and age groups in the 5 years preceding a 1st demyelinating event. Those that showed significant sex-based differences *via* likelihood ratio tests or age-based differences via Bonferroni-adjusted tests are displayed.

A summary of the study results is depicted in Figure 3.

## Discussion

In this population-based study, we identified age- and sex-based differences in health encounters in the 5-years preceding MS onset. We found the effect of MS on overall health-care use in the 5-years prior to a first demyelinating event was generally stronger in males than females and older, vs. younger, individuals. Specifically, the relative rate for males with MS, compared to males without MS, was between 1.2 and 2 times the equivalent relative rate for females for hospitalizations or visits to a physician for disorders of the genitourinary and nervous systems, as well as for “ill-defined” symptoms/signs, and burns. Relative rates among males with MS were also 16% higher for GP visits, and 34% higher for neurologists. Age-specific effects included a 17% higher relative rate among older people with MS for visits to a GP. While younger people with MS had a 15–45% increased relative rate for ophthalmologist and sensory-related visits, and a 18% higher relative rate for cardiovascular-related drugs. Our study provides evidence of sex and age heterogeneity in the 5 years before a first demyelinating event, suggestive of fundamental demographic differences in the MS prodromal presentation.

While there were few other studies with which to compare our findings, the higher relative rates of physician visits and hospitalizations among males and older individuals with MS broadly reflects findings in persons already diagnosed with MS. For example, males with relapsing remitting MS accumulate disability and reach secondary progressive MS faster than females (23), and can also have a higher comorbidity burden at diagnosis (24). Additionally, several studies show that late-onset MS is associated with faster progression to severe disability (23, 25). There is also growing evidence that a higher comorbidity burden is associated with a higher disease activity and more rapid disability progression in persons with MS (26–28). Taken together, our findings suggest that sex and age-differences in the disease trajectory begin even before clinically recognized MS onset.

Our study also suggests that other specific elements of the MS prodrome vary by sex and age. males with MS had up to a 55% higher relative rate for physician visits and prescription fills for genitourinary-related disorders than females with MS. Mood or anxiety disorders and prescriptions for anti-depressants were 14–21% higher among males than females with MS, relative to controls. These findings were all observed in the 5-years before a first demyelinating event. These sex-differences are broadly consistent with studies focused on the prevalent MS population, i.e., after a MS diagnosis. For example, a Canadian study found that males with MS had a higher relative prevalence of anxiety and depression than females at MS diagnosis (24). Further, an Iranian study found that lower urinary tract symptoms, such as intermittent urine stream, hesitancy and straining, were more common in males with MS, though relative comparisons were not made with general population controls (29). Studies have suggested that low testosterone levels are associated with increased MS risk and greater MS disability progression (30, 31). Therefore, it remains possible that low testosterone levels or symptoms suggesting low testosterone such as fatigue, low libido or erectile dysfunction, among men in the prodromal period may lead physicians to prescribe hormonal or genitourinary-related medications. Males with MS were also more likely to have certain comorbidities in our study and these comorbidities may in turn be driving the increased healthcare use observed among males for other conditions in the prodromal phase. Burn injuries may result from MS-related sensory peripheral neuropathy or motor impairment, but they may also be caused by other comorbidities (32).

However, we also found that some aspects of the MS prodrome were more evident for females than males, including a 49% increased relative rate for arthropathies, and 38% higher for IHD. When assessed at, or after a MS diagnosis, studies in Sweden, the UK and Canada similarly found increased relative incidence and prevalence of cardiovascular disease among females vs. males with MS (24, 33, 34). We note that the case definition for IHD includes the diagnostic code for angina, therefore, it is possible that thoracic banding (the “MS hug”) was misattributed to IHD. Although sex differences are not universally observed; a study of Taiwan's registry data found no difference in the incidence of rheumatoid arthritis between males and females diagnosed with MS (35). However, in agreement with our results, a study based in Poland found females with MS were more likely to have health encounters related to the musculoskeletal system before MS diagnosis than males with MS (36). None of these studies examined the period prior to MS onset.

Interestingly, we also found evidence that the relative rate of IHD was more elevated in younger people during the 5-years before MS onset. This is consistent with another study based in Canada that showed 20–44 year-olds already diagnosed with MS had a 55% higher relative prevalence for IHD than 45–59 year-olds (37). Younger people with MS also had a higher relative rate for visits to an ophthalmologist, in line with previous studies showing that younger people are more likely to present with optic neuritis at MS onset (14, 38). Interestingly, this was despite a diagnosis of optic neuritis being defined as a demyelinating event in our study. Prior to a first MS diagnosis, one study also found visual disturbances were more pronounced among younger people with MS (age 20–30) compared to older people with MS (6). Whether some of these findings represent a missed opportunity for the earlier recognition and diagnosis of MS would be of value to determine, and could benefit the person with MS seeking a diagnosis, as well as enhance understanding of the MS prodrome (39).

### Strengths and limitations

Study strengths included: the population-based sampling, maximizing generalizability of findings; the use of prospectively collected data, minimizing recall bias; and the inclusion of a clinical cohort which provided access to the earliest known classical MS symptom onset date. While the time before onset in the clinical cohort arguably reflects the “true” prodrome, the modest size of the cohort likely limited our ability to detect sex and age-based differences in relative rates. Further, our primarily descriptive analyses were unable to consider potential condition-specific confounders or effect modifiers beyond age or sex for each study outcome [e.g., cardiovascular disease is associated with MS (33) and is a risk factor for vertigo (40); anti-depressants might increase fracture risk (41)]. However, we matched cases and controls by sex, birth year and postal code to ensure the MS and control cohorts were comparable on these potential confounders. Finally, we were only able to measure conditions or symptoms that prompted an individual to access the health system, possibly leading to non-differential misclassification. Thus, our findings likely provide conservative estimates of the sex and age-based heterogeneity in the MS prodrome. Finally, future studies extending the 5-year study period are required. It may also be of interest for future studies to examine whether prodromal features are related in any way to the timing of a MS diagnosis, or diagnostic delay. However, a recent study from Denmark found that whilst the time from onset to diagnosis had changed over time (decreased) since the 1950s, it did not differ by sex (42).

### Conclusions

This study provides a comprehensive assessment of sex and age differences in the MS prodrome. Considerable diversity in the presentation of the MS prodrome was observed for males, females and across age groups. These findings provide insight into the heterogeneity of the MS prodrome and support the use of sex and age as predictor variables to improve the accuracy of MS risk prediction models and early detection tools.

## Data availability statement

The data analyzed in this study was obtained from Population Data British Columbia (BC), the following licenses/restrictions apply: Researchers may access these data by working directly with Population Data BC and following Population Data BC processes. Upon approval, data will be provisioned by Population Data BC and released for analysis on the Population Data BC Secure Research Environment (SRE), unless otherwise approved. Requests to access these datasets should be directed to Population Data BC, https://www.popdata.bc.ca/data_access.

## Ethics statement

The studies involving human participants were reviewed and approved by the University of British Columbia Clinical Research Ethics Board and British Columbia Ministry of Health. Written informed consent to participate in this study was provided by the participants' legal guardian/ next of kin.

## Author contributions

Conception and design of the study, acquisition, and analysis of data: FY, JW, EK, FZ, CE, JF, YZ, RM, and HT. Drafting a significant portion of the manuscript or figures: FY, JW, MK, FZ, CE, JF, YZ, RM, and HT. All authors contributed to the article and approved the submitted version.

## Funding

This study was supported by the National Multiple Sclerosis Society (RG5063A4/1; PI: HT). The funding source had no involvement in the study design, the collection, analysis, and interpretation of the data, or in the decision to submit this article for publication. All authors had full access to the data in the study and the corresponding author had the final responsibility for the decision to submit for publication.

## Conflict of interest

Author FY was funded by a Fredrick Banting and Charles Best Canada Graduate Scholarship from the Canadian Institutes of Health Research (CIHR). Author JF receives research funding from: CIHR, Crohn's and Colitis Canada, Research Nova Scotia; consultation and distribution royalties from MAPI Research Trust. Over the past 4 years, Author MK has received consulting fees from Biogen (unrelated to the current project) and participated in Advisory Boards and/or Satellite Symposia of Biogen Inc. Author RM receives research funding from: CIHR, Research Manitoba, Multiple Sclerosis Society of Canada, Multiple Sclerosis Scientific Foundation, Crohn's and Colitis Canada, National Multiple Sclerosis Society, CMSC. She was supported by the Waugh Family Chair in Multiple Sclerosis. She was a co-investigator on studies funded partly by Biogen Idec and Roche (no funds to her, her institution). Author HT has, in the last 5 years, received research support from the Canada Research Chair Program, the National Multiple Sclerosis Society, the Canadian Institutes of Health Research, the Multiple Sclerosis Society of Canada and the Multiple Sclerosis Scientific Research Foundation. In addition, in the last 5 years, has had travel expenses or registration fees prepaid or reimbursed to present at CME conferences from the Consortium of MS Centres (2018), National MS Society (2016, 2018), ECTRIMS/ACTRIMS (2015, 2016, 2017, 2018, 2019, 2020, 2021, 2022), American Academy of Neurology (2015, 2016, 2019). Speaker honoraria are either declined or donated to an MS charity or to an unrestricted grant for use by HT's research group. The remaining authors declare that the research was conducted in the absence of any commercial or financial relationships that could be construed as a potential conflict of interest.

## Publisher's note

All claims expressed in this article are solely those of the authors and do not necessarily represent those of their affiliated organizations, or those of the publisher, the editors and the reviewers. Any product that may be evaluated in this article, or claim that may be made by its manufacturer, is not guaranteed or endorsed by the publisher.

## Author disclaimer

All inferences, opinions, and conclusions drawn in this publication are those of the author(s), and do not reflect the opinions or policies of the Data Steward(s).
