# Supplementary material for: Sex and age differences in the Multiple Sclerosis prodrome
Source: Front Neurol. 2022 Nov 3;13:1017492. doi: 10.3389/fneur.2022.1017492 (PMC9668896; doi:10.3389/fneur.2022.1017492)
Supplement: Supplementary file 1 [file Data_Sheet_1.docx]

**Supplemental material**

**Table S1:** Multiple sclerosis-specific and demyelinating disease related International Classification of Diseases-9/10 (ICD-9/10) codes and Drug Identification Numbers used to identify people with MS and their respective index date (i.e., first recorded demyelinating event)

|  | **ICD-9** | **ICD-10** | **Drug Identification Number** |
| --- | --- | --- | --- |
| **MS specific diagnosis** | | | |
| Multiple sclerosis | 340 | G35 | N/A |
| **Demyelinating disease** | | | |
| Optic neuritis | 377.3 | H46 | N/A |
| Acute transverse myelitis | 323.82  341.2 | G37.3 | N/A |
| Acute disseminated encephalomyelitis | 323 | G36.9 | N/A |
| Demyelinating disease of CNS unspecified | 341.9 | G37.8 | N/A |
| Other acute disseminated demyelination |  | G36 | N/A |
| Neuromyelitis optica | 341.0 | G36.0 | N/A |
| **MS specific prescription,**  **brand name (generic)** | | | |
| Betaseron (IFNB-1b) | N/A | N/A | 02169649 |
| Extavia (IFNB-1b) | N/A | N/A | 02337819 |
| Avonex (IFNB-1a) | N/A | N/A | 02237770  02269201 |
| Rebif (IFNB-1a) | N/A | N/A | 02281708  02277492  02237317  02237319  02237320  02318253  02318261 |
| Copaxone (glatiramer acetate) | N/A | N/A | 02233014  02245619 |
| Tysabri (natalizumab) | N/A | N/A | 02286386 |
| Gilenya (fingolimod) | N/A | N/A | 02365480 |
| Tecfidera  (dimethyl fumarate) | N/A | N/A | 02404508 |
| Aubagio (teriflunomide) | N/A | N/A | 02416328 |
| Lemtrada (alemtuzumab) | N/A | N/A | 02418320 |

**Table S2:** Diagnostic codes for the International Classification of Diseases (ICD) -9/10 chapters

| **ICD Chapters** | **Diagnostic Codes** | **Brief Description**‡ |
| --- | --- | --- |
| Certain infectious and parasitic diseases | ICD-9: 001 – 139  ICD-10: A00 – B99 | These include bacterial, viral, parasitic, and fungal infections. Some organ-specific infections, such as cystitis and pneumonia, are included in separate chapters. |
| Neoplasms | ICD-9: 140 – 239 ICD-10: C00 – D49 | These include malignant and benign neoplasms. |
| Endocrine, Nutritional and Metabolic Diseases, and Immunity Disorders | ICD-9: 240 – 279 ICD-10: E00 – E89 | These include: disorders of the endocrine glands, such as thyroiditis and diabetes mellitus; nutritional deficiencies; and other metabolic/immunity disorders. |
| Diseases of The Blood and Blood-Forming Organs | ICD-9: 280– 289 ICD-10: D50 – D89 | These include: anemias, such as iron or B12 deficiency; hemolytic and aplastic anemias; and diseases of the white blood cells |
| Mental, Behavioral and Neurodevelopmental disorders | ICD-9: 290 – 319  ICD-10: F00 – F99 | These include: mood disorders, such as depression and bipolar disorder; non-mood psychotic disorders; anxiety; intellectual disabilities; developmental disorders; and emotional disorders |
| Diseases of the nervous system | ICD-9: 320 – 359  ICD-10: G00 – G99 | These include: inflammatory disease of the central nervous system, such as meningitis; extrapyramidal and movement disorders, such as Parkinson’s disease and dystonia; neurodegenerative disease; polyneuropathies; and paroxysmal disorders, such as epilepsy and migraine. Demyelinating diseases were excluded. |
| Diseases of the sense organs | ICD-9: 360 – 389  ICD-10: H00-H95 | These include: disorders of the eye, such as visual disturbances, disorders of the lens, retina, or eyelid; and disorders of the ear, such as hearing loss, vertiginous syndromes, and otitis media/externa. Disorders of the optic nerve were excluded. |
| Diseases of the circulatory system | ICD-9: 390 – 459  ICD-10: I00 – I99 | These include: hypertensive diseases; heart diseases, such as ischemic or pulmonary heart disease; cerebrovascular disease, such as the occlusion of cerebral arteries; and disease of the veins and arteries, such as atherosclerosis and varicose veins. |
| Diseases of the respiratory system | ICD-9: 460 – 519  ICD-10: J00 – J99 | These include: respiratory infections, such as influenza and pneumonia; chronic respiratory disease, such as asthma and bronchitis; and diseases of the  pleura. |
| Diseases of the digestive system | ICD-9: 520 – 579  ICD-10: K00 – K95 | These include: disease of the intestines, such as irritable bowel syndrome; diseases of the liver, such as hepatitis and inflammatory liver disease; disorders of the gallbladder, such as acute pancreatitis; ulcers; and hernia. |
| Diseases of the genitourinary system | ICD-9: 580 – 629  ICD-10: N00 – N99 | These include: disorders of the urinary tract, such as chronic kidney disease, nephritis, and cystitis; and disease of the genital organs, such as sexual dysfunction, disorders of the prostate, pelvic inflammatory disorders, absent or excessive menstruation; and disorders of the breast, such as unspecified lump in breast. |
| Diseases of the skin and subcutaneous  tissues | ICD-9: 680 – 709  ICD-10: L00 – L99 | These include: infections of the skin; dermatitis and eczema; erythema; and papulosquamous disorders, such as psoriasis. |
| Diseases of the musculoskeletal system and connective tissues | ICD-9: 710 – 739  ICD-10: M00 – M99 | These include: arthropathies; dorsopathies; osteopathies; spondylopathies; osteoarthritis; and  disorders of the muscles. |
| Congenital anomalies | ICD-9: 740-759  ICD-10: Q00–Q99 | These include congenital malformations of the nervous system, circulatory system, respiratory system, genital organs, urinary system, eyes, and ears. |
| Symptoms, Signs, And Ill-Defined  Conditions | ICD-9: 780 – 799  ICD-10: R00 – R99 | These include symptoms and signs involving the circulatory, respiratory, nervous, musculoskeletal, genitourinary system. As well as, symptoms involving cognition, emotional state, behaviour, speech. Abnormal findings on examination of blood,  urine or other body fluids. |
| Injury and poisoning  External causes of morbidity complication of surgical and medical care | ICD-9: 800-999  ICD-9: E800 – E999  ICD-10: S00 – T98  ICD-10: V00-Y99 | These include: injuries and burns; poisoning; accidents; slipping and falls; and misadventures during surgical and medical care. |

**‡** Codes related to pregnancy, childbirth and the puerperium (ICD-9: 630-679/ ICD-10: O00–O99), and conditions originating in the perinatal period (ICD-9: 760-779/ ICD-10: P00–P96) were excluded.

**Table S3:** Physician Specialties

| **Physician speciality**‡ |
| --- |
| Primary care provider / general practice |
| Ophthalmologist and otolaryngologist |
| Physiatrist (i.e. physical medicine & rehabilitation specialist) |
| Psychiatrist (also includes neuropsychiatrist) |
| Internal medicine physician (also includes rheumatologist, cardiologist, and gastroenterology) |
| Neurologist |
| Urologist |

**‡** Visits to an obstetrician and gynecologist were excluded.

**Table S4**: Drug classes based on the Anatomical Therapeutic Chemical Classification System Level 1

| **Anatomical group (ATC level 1)** | **ATC Code** | **Brief Description** |
| --- | --- | --- |
| Alimentary tract and metabolism | A | These include: drugs for gastrointestinal disorders; anti-nauseants; bile and liver therapy; antidiarrheals; vitamins; and drugs used in diabetes. |
| Blood and blood forming organs | B | These include: antithrombotic agents; anti-hemorrhagics; anti-anemic preparations; and blood substitutes. |
| Cardiovascular system | C | These include: anti-hypertensives; diuretics; vasodilators; and lipid modifying agents. |
| Dermatologicals | D | These include: antifungals; anti- psoriatics; antibiotics and corticosteroids for dermatological use; preparations for treatment of wounds and ulcers; and anti-acne preparations. |
| Genito-urinary system and sex hormones | G | These include: gynecological anti-infectives; sex hormones, modulators of the genital system; and urologicals |
| Systemic hormonal preparations, excluding sex hormones and insulins | H | These include: pituitary and hypothalamic hormones; corticosteroids; thyroid therapy; pancreatic hormones; and calcium homeostasis |
| Anti-infectives for systemic use | J | These include: anti-bacterials, anti-mycotics, anti-mycobacterials and antivirals for systemic use; immunoglobulins; and vaccines |
| Antineoplastic and immunomodulating agents | L | These include: anti-neoplastic agents; endocrine therapy; immunostimulants; and immunosuppressants. MS specific drugs were excluded. |
| Musculo-skeletal system | M | These include: anti-inflammatory and anti-rheumatic products; topical products for joint/muscular pain; muscle relaxants; anti-gout preparations; and drugs for treatment of bone disease. |
| Nervous system | N | These include: anesthetics; analgesics; anti-epileptics; anti-parkinsons drugs; and psycholeptics; psychoanaleptics. MS specific drugs were excluded. |
| Anti-parasitic products, insecticides and repellents | P | These include: anti-protozoals; anti-helmintics; and ectoparasticides |
| Respiratory system | R | These include: nasal and throat preparations; drugs for obstructive airway diseases; cough and cold preparations; and antihistamines for systemic use. |
| Sensory organs | S | These include ophthalmologicals and otologicals. |
| Various | V | These include: allergens; diagnostics agents; nutrients; contrast media; radiopharmaceuticals; and surgical dressings |

**Table S5:** International Classification of Diseases (ICD)-9 sub-chapter groupings

| **ICD-9 Chapters** | **ICD-9 Sub-Chapter**‡ | **ICD-9 Codes** |
| --- | --- | --- |
| Certain infectious and parasitic diseases | Intestinal Infectious Diseases | 001-009 |
|  | Tuberculosis | 010-018 |
|  | Zoonotic Bacterial Diseases | 020-027 |
|  | Other Bacterial Diseases | 030-041 |
|  | Human Immunodeficiency Virus | 042-042 |
|  | Poliomyelitis and Other Non-Arthropod-Borne Viral Diseases of Central Nervous System | 045-049 |
|  | Viral Diseases Accompanied by Exanthem | 050-059 |
|  | Arthropod-Borne Viral Diseases | 060-066 |
|  | Other Diseases Due to Viruses and Chlamydiae | 070-079 |
|  | Rickettsioses and Other Arthropod-Borne Diseases | 080-088 |
|  | Syphilis and Other Venereal Diseases | 090-099 |
|  | Other Spirochetal Diseases | 100-104 |
|  | Mycoses | 110-118 |
|  | Helminthiases | 120-129 |
|  | Other Infectious and Parasitic Diseases | 130-136 |
|  | Late Effects of Infectious and Parasitic Diseases | 137-139 |
| Neoplasms | Malignant Neoplasm of Lip, Oral Cavity, and Pharynx | 140-149 |
|  | Malignant Neoplasm of Digestive Organs and Peritoneum | 150-159 |
|  | Malignant Neoplasm of Respiratory and Intrathoracic Organs | 160-165 |
|  | Malignant Neoplasm of Bone, Connective Tissue, Skin, and Breast | 170-176 |
|  | Malignant Neoplasm of Genitourinary Organs | 179-189 |
|  | Malignant Neoplasm of Other and Unspecified Sites | 190-199 |
|  | Malignant Neoplasm of Lymphatic and Hematopoietic Tissue | 200-209 |
|  | Benign Neoplasms | 210-229 |
|  | Carcinoma in Situ | 230-234 |
|  | Neoplasms of Uncertain Behavior | 235-238 |
|  | Neoplasms of Unspecified Nature | 239-239 |
| Endocrine, Nutritional and Metabolic Diseases, and Immunity Disorders | Disorders of Thyroid Gland | 240-246 |
|  | Diseases of Other Endocrine Glands | 249-259 |
|  | Nutritional Deficiencies | 260-269 |
|  | Other Metabolic Disorders and Immunity Disorders | 270-279 |
| Diseases of The Blood and Blood-Forming Organs | Iron Deficiency Anemias | 280 |
|  | Other Deficiency Anemias | 281 |
|  | Hereditary Hemolytic Anemias | 282 |
|  | Acquired Hemolytic Anemias | 283 |
|  | Aplastic Anemia and Other Bone Marrow Failure Syndromes | 284 |
|  | Other and Unspecified Anemias | 285 |
|  | Coagulation Defects | 286 |
|  | Purpura and Other Hemorrhagic Conditions | 287 |
|  | Diseases of White Blood Cells | 288 |
|  | Other Diseases of Blood and Blood-Forming Organs | 289 |
| Mental, Behavioral and Neurodevelopmental disorders | Organic Psychotic Conditions | 290-294 |
|  | Other Psychoses | 295-299 |
|  | Neurotic Disorders, Personality Disorders, And Other Nonpsychotic Mental Disorders | 300-316 |
|  | Intellectual Disabilities | 317-319 |
| Diseases of the nervous system | Inflammatory Diseases of The Central Nervous System | 320-327 |
|  | Hereditary and Degenerative Diseases of The Central Nervous System | 330-337 |
|  | Pain | 338-338 |
|  | Other Headache Syndromes | 339-339 |
|  | Other Disorders of The Central Nervous System | 340-349 |
|  | Disorders of The Peripheral Nervous System | 350-359 |
| Diseases of the sense organs | Disorders of The Eye and Adnexa | 360-379 |
|  | Diseases of The Ear and Mastoid Process | 380-389 |
| Diseases of the circulatory system | Acute Rheumatic Fever | 390-392 |
|  | Chronic Rheumatic Heart Disease | 393-398 |
|  | Hypertensive Disease | 401-405 |
|  | Ischemic Heart Disease | 410-414 |
|  | Diseases of Pulmonary Circulation | 415-417 |
|  | Other Forms of Heart Disease | 420-429 |
|  | Cerebrovascular Disease | 430-438 |
|  | Diseases of Arteries, Arterioles, and Capillaries | 440-449 |
|  | Diseases of Veins and Lymphatics, and Other Diseases of Circulatory System | 451-459 |
| Diseases of the respiratory system | Acute Respiratory Infections | 460-466 |
|  | Other Diseases of Upper Respiratory Tract | 470-478 |
|  | Pneumonia and Influenza | 480-488 |
|  | Chronic Obstructive Pulmonary Disease and Allied Conditions | 490-496 |
|  | Pneumoconioses and Other Lung Diseases due to External Agents | 500-508 |
|  | Other Diseases of Respiratory System | 510-519 |
| Diseases of the digestive system | Diseases of Oral Cavity, Salivary Glands, And Jaws | 520-529 |
|  | Diseases of Esophagus, Stomach, And Duodenum | 530-539 |
|  | Appendicitis | 540-543 |
|  | Hernia of Abdominal Cavity | 550-553 |
|  | Noninfective Enteritis and Colitis | 555-558 |
|  | Other Diseases of Intestines and Peritoneum | 560-569 |
|  | Other Diseases of Digestive System | 570-579 |
| Diseases of the genitourinary system | Nephritis, Nephrotic Syndrome, and Nephrosis | 580-589 |
|  | Other Diseases of Urinary System | 590-599 |
|  | Diseases of Male Genital Organs | 600-608 |
|  | Disorders of Breast | 610-612 |
|  | Inflammatory Disease of Female Pelvic Organs | 614-616 |
|  | Other Disorders of Female Genital Tract | 617-629 |
| Diseases of the skin and subcutaneous  tissues | Infections of Skin and Subcutaneous Tissue | 680-686 |
|  | Other Inflammatory Conditions of Skin and Subcutaneous Tissue | 690-698 |
|  | Other Diseases of Skin and Subcutaneous Tissue | 700-709 |
| Diseases of the musculoskeletal system and connective tissues | Arthropathies And Related Disorders | 710-719 |
|  | Dorsopathies | 720-724 |
|  | Rheumatism, Excluding the Back | 725-729 |
|  | Osteopathies, Chondropathies, And Acquired Musculoskeletal Deformities | 730-739 |
| Congenital anomalies | Congenital anomalies | 740-759 |
| Symptoms, Signs, And Ill-Defined  Conditions | Symptoms | 780-789 |
|  | Nonspecific Abnormal Findings | 790-796 |
|  | Ill-Defined and Unknown Causes of Morbidity and Mortality | 797-799 |
| Injury and poisoning  External causes of morbidity complication of surgical and medical care | Fracture of Skull | 800-804 |
|  | Fracture of Spine and Trunk | 805-809 |
|  | Fracture of Upper Limb | 810-819 |
|  | Fracture of Lower Limb | 820-829 |
|  | Dislocation | 830-839 |
|  | Sprains and Strains of Joints and Adjacent Muscles | 840-848 |
|  | Intracranial Injury, Excluding Those with Skull Fracture | 850-854 |
|  | Internal Injury of Chest, Abdomen, And Pelvis | 860-869 |
|  | Open Wound of Head, Neck, And Trunk | 870-879 |
|  | Open Wound of Upper Limb | 880-887 |
|  | Open Wound of Lower Limb | 890-897 |
|  | Injury to Blood Vessels | 900-904 |
|  | Late Effects of Injuries, Poisonings, Toxic Effects, And Other External Causes | 905-909 |
|  | Superficial Injury | 910-919 |
|  | Contusion with Intact Skin Surface | 920-924 |
|  | Crushing Injury | 925-929 |
|  | Effects of Foreign Body Entering Through Orifice | 930-939 |
|  | Burns | 940-949 |
|  | Injury to Nerves and Spinal Cord | 950-957 |
|  | Certain Traumatic Complications and Unspecified Injuries | 958-959 |
|  | Poisoning by Drugs, Medicinals and Biological Substances | 960-979 |
|  | Toxic Effects of Substances Chiefly Nonmedicinal as To Source | 980-989 |
|  | Other and Unspecified Effects of External Causes | 990-995 |
|  | Complications of Surgical and Medical Care, Not Elsewhere Classified | 996-999 |

**‡** Codes related to pregnancy, childbirth and the puerperium (ICD-9: 630-679/ ICD-10: O00–O99), and conditions originating in the perinatal period (ICD-9: 760-779/ ICD-10: P00–P96) were excluded.

**Table S6:** Drug classes based on the Anatomical Therapeutic Chemical Classification System Level 3

| **ATC Level 1** | **ATC Level 3** | **ATC Level 3 Codes** |
| --- | --- | --- |
| Alimentary tract and metabolism | Stomatological Preparations | A01A |
|  | Antacids | A02A |
|  | Drugs For Peptic Ulcer and Gastro-Oesophageal Reflux Disease (Gord) | A02B |
|  | Antiflatulents | A02D |
|  | Drugs for Functional Bowel Disorders | A03A |
|  | Belladonna and Derivatives, Plain | A03B |
|  | Antispasmodics in Combination with Psycholeptics | A03C |
|  | Antispasmodics and Anticholinergics in Combination with other Drugs | A03E |
|  | Propulsives | A03F |
|  | Antiemetics and Antinauseants | A04A |
|  | Bile Therapy | A05A |
|  | Laxatives | A06A |
|  | Intestinal Antiinfectives | A07A |
|  | Intestinal Adsorbents | A07B |
|  | Electrolytes with Carbohydrates | A07C |
|  | Antipropulsives | A07D |
|  | Intestinal Antiinflammatory Agents | A07E |
|  | Antidiarrheal Microorganisms | A07F |
|  | Antiobesity Preparations, excl. Diet Products | A08A |
|  | Digestives, incl. Enzymes | A09A |
|  | Insulins And Analogues | A10A |
|  | Blood Glucose Lowering Drugs, Excl. Insulins | A10B |
|  | Multivitamins, Combinations | A11A |
|  | Multivitamins, Plain | A11B |
|  | Vitamin A and D, Incl. Combinations of the two | A11C |
|  | Vitamin B1, Plain and In Combination with Vitamin B6 and B12 | A11D |
|  | Vitamin B-Complex, incl. combinations | A11E |
|  | Ascorbic Acid (Vitamin C), Incl. Combinations | A11G |
|  | Other Plain Vitamin Preparations | A11H |
|  | Other vitamin products, combinations | A11J |
|  | Calcium | A12A |
|  | Potassium | A12B |
|  | Other Mineral Supplements | A12C |
|  | Anabolic Steroids | A14A |
|  | Other Alimentary Tract and Metabolism Products | A16A |
| Blood and blood forming organs | Antithrombotic Agents | B01A |
|  | Antifibrinolytics | B02A |
|  | Vitamin K and Other Hemostatics | B02B |
|  | Iron Preparations | B03A |
|  | Vitamin B12 and Folic Acid | B03B |
|  | Other Antianemic Preparations | B03X |
|  | Blood And Related Products | B05A |
|  | I.V. Solutions | B05B |
|  | Irrigating Solutions | B05C |
|  | I.V. Solution Additives | B05X |
|  | Other Hematological Agents | B06A |
| Cardiovascular system | Cardiac Glycosides | C01A |
|  | Antiarrhythmics, Class I and III | C01B |
|  | Cardiac Stimulants Excl. Cardiac Glycosides | C01C |
|  | Vasodilators Used in Cardiac Diseases | C01D |
|  | Other Cardiac Preparations | C01E |
|  | Antiadrenergic Agents, Centrally Acting | C02A |
|  | Antiadrenergic Agents, Peripherally Acting | C02C |
|  | Arteriolar Smooth Muscle, Agents Acting on | C02D |
|  | Other Antihypertensives | C02K |
|  | Antihypertensives and Diuretics in Combination | C02L |
|  | Low-Ceiling Diuretics, Thiazides | C03A |
|  | Low-Ceiling Diuretics, Excl. Thiazides | C03B |
|  | High-Ceiling Diuretics | C03C |
|  | Potassium-Sparing Agents | C03D |
|  | Diuretics And Potassium-Sparing Agents in Combination | C03E |
|  | Other Diuretics | C03X |
|  | Peripheral Vasodilators | C04A |
|  | Agents For Treatment of Hemorrhoids And Anal Fissures For Topical Use | C05A |
|  | Antivaricose Therapy | C05B |
|  | Beta Blocking Agents | C07A |
|  | Beta Blocking Agents and Thiazides | C07B |
|  | Beta Blocking Agents and Other Diuretics | C07C |
|  | Selective Calcium Channel Blockers with Mainly Vascular Effects | C08C |
|  | Selective Calcium Channel Blockers with Direct Cardiac Effects | C08D |
|  | Ace Inhibitors, Plain | C09A |
|  | Ace Inhibitors, Combinations | C09B |
|  | Angiotensin II Antagonists, Plain | C09C |
|  | Angiotensin II Antagonists, Combinations | C09D |
|  | Other Agents Acting on The Renin-Angiotensin System | C09X |
|  | Lipid Modifying Agents, Plain | C10A |
|  | Lipid Modifying Agents, Combinations | C10B |
| Dermatologicals | Antifungals for Topical Use | D01A |
|  | Antifungals for Systemic Use | D01B |
|  | Emollients And Protectives | D02A |
|  | Protectives Against UV-Radiation | D02B |
|  | Cicatrizants | D03A |
|  | Enzymes | D03B |
|  | Antipruritics, Incl. Antihistamines, Anesthetics, Etc. | D04A |
|  | Antipsoriatics for Topical Use | D05A |
|  | Antipsoriatics for Systemic Use | D05B |
|  | Antibiotics For Topical Use | D06A |
|  | Chemotherapeutics For Topical Use | D06B |
|  | Corticosteroids, Plain | D07A |
|  | Corticosteroids, Combinations with Antibiotics | D07C |
|  | Corticosteroids, Other Combinations | D07X |
|  | Antiseptics And Disinfectants | D08A |
|  | Medicated Dressings | D09A |
|  | Anti-Acne Preparations for Topical Use | D10A |
|  | Anti-Acne Preparations for Systemic Use | D10B |
|  | Other Dermatological Preparations | D11A |
| Genito-urinary system and sex hormones | Antiinfectives and Antiseptics, Excl. Combinations with Corticosteroids | G01A |
|  | Oxytocics | G02A |
|  | Contraceptives For Topical Use | G02B |
|  | Other Gynecologicals | G02C |
|  | Hormonal Contraceptives for Systemic Use | G03A |
|  | Androgens | G03B |
|  | Estrogens | G03C |
|  | Progestogens | G03D |
|  | Androgens and Female Sex Hormones in Combination | G03E |
|  | Progestogens and Estrogens in Combination | G03F |
|  | Gonadotropins and Other Ovulation Stimulants | G03G |
|  | Antiandrogens | G03H |
|  | Other Sex Hormones and Modulators of The Genital System | G03X |
|  | Other Urologicals, Incl. Antispasmodics | G04B |
|  | Drugs Used In Benign Prostatic Hypertrophy | G04C |
| Systemic hormonal preparations, excluding sex hormones and insulins | Anterior Pituitary Lobe Hormones and Analogues | H01A |
|  | Posterior Pituitary Lobe Hormones | H01B |
|  | Hypothalamic Hormones | H01C |
|  | Corticosteroids For Systemic Use, Plain | H02A |
|  | Corticosteroids For Systemic Use, Combinations | H02B |
|  | Thyroid Preparations | H03A |
|  | Antithyroid Preparations | H03B |
|  | Glycogenolytic Hormones | H04A |
|  | Parathyroid Hormones and Analogues | H05A |
|  | Anti-Parathyroid Agents | H05B |
| Anti-infectives for systemic use | Tetracyclines | J01A |
|  | Amphenicols | J01B |
|  | Beta-Lactam Antibacterials, Penicillins | J01C |
|  | Other Beta-Lactam Antibacterials | J01D |
|  | Sulfonamides And Trimethoprim | J01E |
|  | Macrolides, Lincosamides and Streptogramins | J01F |
|  | Aminoglycoside Antibacterials | J01G |
|  | Quinolone Antibacterials | J01M |
|  | Combinations Of Antibacterials | J01R |
|  | Other Antibacterials | J01X |
|  | Antimycotics For Systemic Use | J02A |
|  | Drugs For Treatment of Tuberculosis | J04A |
|  | Drugs For Treatment of Lepra | J04B |
|  | Direct Acting Antivirals | J05A |
|  | Immune Sera | J06A |
|  | Immunoglobulins | J06B |
|  | Bacterial Vaccines | J07A |
|  | Viral Vaccines | J07B |
|  | Bacterial And Viral Vaccines, Combined | J07C |
| Antineoplastic and immunomodulating agents | Alkylating Agents | L01A |
|  | Antimetabolites | L01B |
|  | Plant Alkaloids and Other Natural Products | L01C |
|  | Cytotoxic Antibiotics and Related Substances | L01D |
|  | Other Antineoplastic Agents | L01X |
|  | Hormones And Related Agents | L02A |
|  | Hormone Antagonists and Related Agents | L02B |
|  | Immunostimulants | L03A |
|  | Immunosuppressants | L04A |
| Musculo-skeletal system | Antiinflammatory And Antirheumatic Products, Non-Steroids | M01A |
|  | Specific Antirheumatic Agents | M01C |
|  | Topical Products for Joint and Muscular Pain | M02A |
|  | Muscle Relaxants, Peripherally Acting Agents | M03A |
|  | Muscle Relaxants, Centrally Acting Agents | M03B |
|  | Muscle Relaxants, Directly Acting Agents | M03C |
|  | Antigout Preparations | M04A |
|  | Drugs Affecting Bone Structure and Mineralization | M05B |
|  | Other Drugs for Disorders of The Musculo-Skeletal System | M09A |
| Nervous system | Anesthetics, General | N01A |
|  | Anesthetics, Local | N01B |
|  | Opioids | N02A |
|  | Other Analgesics and Antipyretics | N02B |
|  | Antimigraine Preparations | N02C |
|  | Antiepileptics | N03A |
|  | Anticholinergic Agents | N04A |
|  | Dopaminergic Agents | N04B |
|  | Antipsychotics | N05A |
|  | Anxiolytics | N05B |
|  | Hypnotics And Sedatives | N05C |
|  | Antidepressants | N06A |
|  | Psychostimulants, Agents Used for Adhd and Nootropics | N06B |
|  | Psycholeptics And Psychoanaleptics in Combination | N06C |
|  | Anti-Dementia Drugs | N06D |
|  | Parasympathomimetics | N07A |
|  | Drugs Used in Addictive Disorders | N07B |
|  | Antivertigo Preparations | N07C |
|  | Other Nervous System Drugs | N07X |
| Anti-parasitic products, insecticides and repellents | Agents Against Amoebiasis and Other Protozoal Diseases | P01A |
|  | Antimalarials | P01B |
|  | Agents Against Leishmaniasis and Trypanosomiasis | P01C |
|  | Antitrematodals | P02B |
|  | Antinematodal Agents | P02C |
|  | Ectoparasiticides, Incl. Scabicides | P03A |
| Respiratory system | Decongestants and Other Nasal Preparations for Topical Use | R01A |
|  | Nasal Decongestants for Systemic Use | R01B |
|  | Throat Preparations | R02A |
|  | Adrenergics, Inhalants | R03A |
|  | Other Drugs for Obstructive Airway Diseases, Inhalants | R03B |
|  | Adrenergics For Systemic Use | R03C |
|  | Other Systemic Drugs for Obstructive Airway Diseases | R03D |
|  | Expectorants, Excl. Combinations with Cough Suppressants | R05C |
|  | Cough Suppressants, Excl. Combinations with Expectorants | R05D |
|  | Cough Suppressants and Expectorants, Combinations | R05F |
|  | Other cold preparations | R05X |
|  | Antihistamines For Systemic Use | R06A |
|  | Other Respiratory System Products | R07A |
| Sensory Organs | Antiinfectives | S01A |
|  | Antiinflammatory Agents | S01B |
|  | Antiinflammatory Agents and Antiinfectives in Combination | S01C |
|  | Antiglaucoma Preparations and Miotics | S01E |
|  | Mydriatics and Cycloplegics | S01F |
|  | Decongestants and Antiallergics | S01G |
|  | Local Anesthetics | S01H |
|  | Diagnostic Agents | S01J |
|  | Surgical Aids | S01K |
|  | Ocular Vascular Disorder Agents | S01L |
|  | Other Ophthalmologicals | S01X |
|  | Antiinfectives | S02A |
|  | Corticosteroids And Antiinfectives in Combination | S02C |
|  | Other Otologicals | S02D |
|  | Antiinfectives | S03A |
|  | Corticosteroids | S03B |
|  | Corticosteroids And Antiinfectives in Combination | S03C |

**Table S7:** Comorbidities identified in the 5 years before the index date in the health administrative cohort

| **Morbidity** | **Years of data** | **Algorithm: number and type of encounters** | **ICD9 codes** | **ICD10 codes** | **ATC codes** |
| --- | --- | --- | --- | --- | --- |
| ***Mental health*** |  |  |  |  |  |
| Any mood or anxiety disorder^1^ | 5 | ≥1 H or ≥5P | 300.0, 300.2, 296.0, 296.1, 296.04, 296.14, 296.4, 296.44, 296.5, 296.54, 296.6, 296.7, 296.8, 296.2, 296.3, 298.0, 300.4, 311 | F40, F41, F31, F32, F33, F34 | N/A |
| Schizophrenia^1^ | 2 | ≥1H or ≥2P | 295 | F20, F25 | N/A |
| ***Vascular*** |  |  |  |  |  |
| Hypertension^2^ | 2 | ≥1H or ≥2P | 401-405 | I10-I13, I15 | N/A |
| Hyperlipidemia^2^ | 5 | ≥1H or ≥2P | 272 | E780, E782, E784, E785 | N/A |
| Diabetes^2^ | 5 | ≥1H or ≥2P | 250 | E10 -E14 | N/A |
| Ischemic heart disease^3^ | 5 | ≥1 H or ≥2P | 410–414 | I20–I25 | N/A |
| ***Other neurologic*** |  |  |  |  |  |
| Epilepsy^4^ | 3 | ≥1 H OR ≥2P | 345 | G40, G41 | N/A |
| ***Other*** |  |  |  |  |  |
| Fibromyalgia^5^ | 3 | ≥5 (H or P) | 729.1 | M79.7 | N/A |
| Irritable bowel syndrome^4^ | 2 | ≥1 H or ≥2P | 564.1 | K58 | N/A |
| Chronic lung disease^4^ | 5 | ≥1H or ≥2P | 493, 491, 492, 496 | J45, J46, J40, J42, J43, J44 | N/A |
| ***Autoimmune*** |  |  |  |  |  |
| Inflammatory bowel disease^4^ | All available data | ≥5 (H or P) | 555, 556 | K50, K51 | N/A |
| Thyroid disorder | 1 | ≥1H or ≥1P or | 242, 376.21, | E05.0 | H03AA01 |
| (Autoimmune) ^6^* |  | ≥1Rx | 376.22, 245, | E06.3 | H03AA05 H03BA02 |
|  |  |  |  |  | H03BA03 |
|  |  |  |  |  | H03BB02 |
|  |  |  |  |  | H03CA |
| Psoriasis^7^ |  |  | 696.0, 696.1 | L40, M07.0- | D05AC01 |
|  |  |  |  | M07.3 | D05AC51 |
|  |  |  |  |  | D05AX |
|  | 3 | ≥2 (H or P or Rx) |  |  | D05AX02 D05AX03 D05AX05 |
|  |  |  |  |  | D05AX52 |
|  |  |  |  |  | D05BA03 |
|  |  |  |  |  | D05BB02 |
| ***Neurological*** |  |  |  |  |  |
| Migraine^4^ | 2 | ≥2 (H or P or Rx) | 345, 625.4 | G43 | N02CA N02CC N02CX |

Key: ICD: International Classification of Diseases; ATC: Anatomical Therapeutic Chemical; H: hospital;

P: physician visits; Rx: prescription

^*^ People who were either diagnosed with thyroid neoplasm, underwent thyroid surgery, or filled a prescription for lithium (ATC= N05AN01) pre-‘onset’ of a thyroid condition were not included here

References

1. Marrie RA, Fisk JD, Yu BN, et al. Mental comorbidity and multiple sclerosis: validating administrative data to support population-based surveillance. BMC Neurol. 2013;13(1):16.

2. Marrie RA, Yu BN, Leung S, et al. Rising prevalence of vascular comorbidities in multiple sclerosis: validation of administrative definitions for diabetes, hypertension, and hyperlipidemia. Mult Scler J. 2012;18(9):1310-1319.

3. Marrie RA, Yu BN, Leung S, et al. Prevalence and incidence of ischemic heart disease in multiple sclerosis: A population-based validation study. Mult Scler Relat Disord. 2013;2(4):355-361.

4. Marrie RA, Yu BN, Leung S, et al. The Utility of Administrative Data for Surveillance of Comorbidity in Multiple Sclerosis: A Validation Study. Neuroepidemiology. 2013;40(2):85-92.

5. Marrie RA, Yu BN, Leung S, Elliott L, Warren S, Wolfson C, et al. The incidence and prevalence of fibromyalgia are higher in multiple sclerosis than the general population: A population-based study. Multiple Sclerosis and Related Disorders. 2012 Oct 1;1(4):162–7.

6. Marrie RA, Yu BN, Leung S, et al. The Incidence and Prevalence of Thyroid Disease Do Not Differ in the Multiple Sclerosis and General Populations: A Validation Study Using Administrative Data. Neuroepidemiology. 2012;39(2):135-142.

7. Marrie RA, Patten SB, Tremlett H, Wolfson C, Leung S, Fisk JD. Increased incidence and prevalence of psoriasis in multiple sclerosis. Mult Scler Relat Disord. 2017;13:81-86.


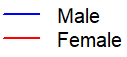

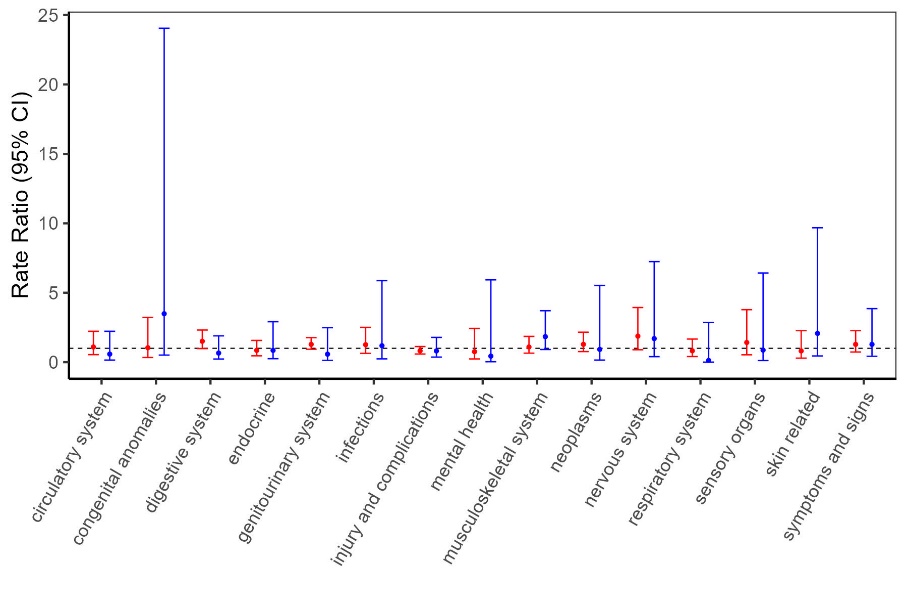

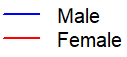

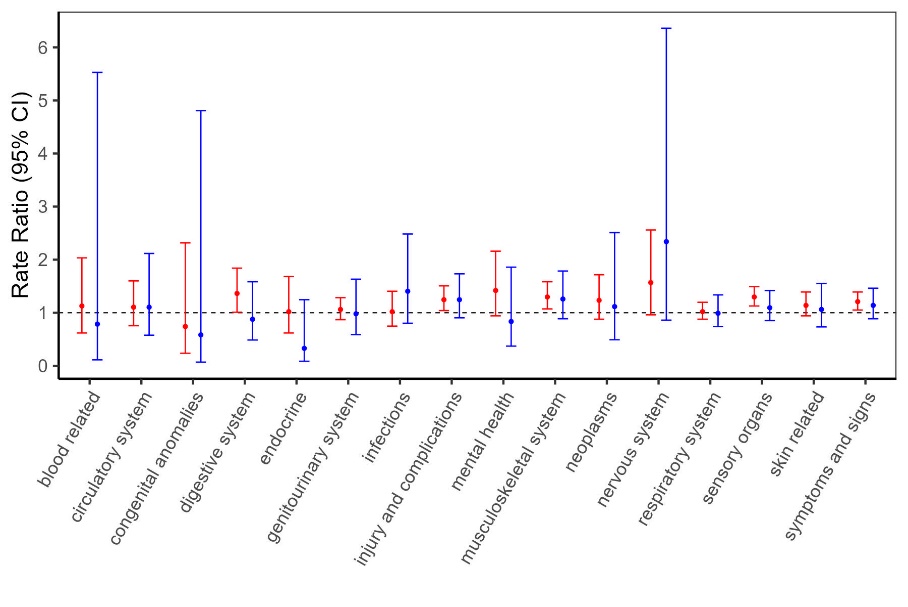
 a) b)


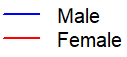

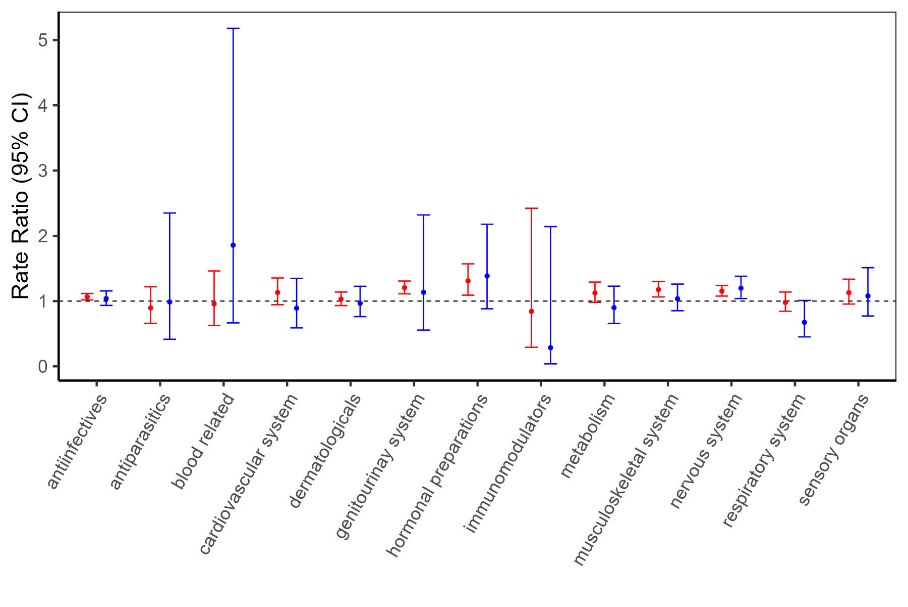

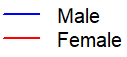

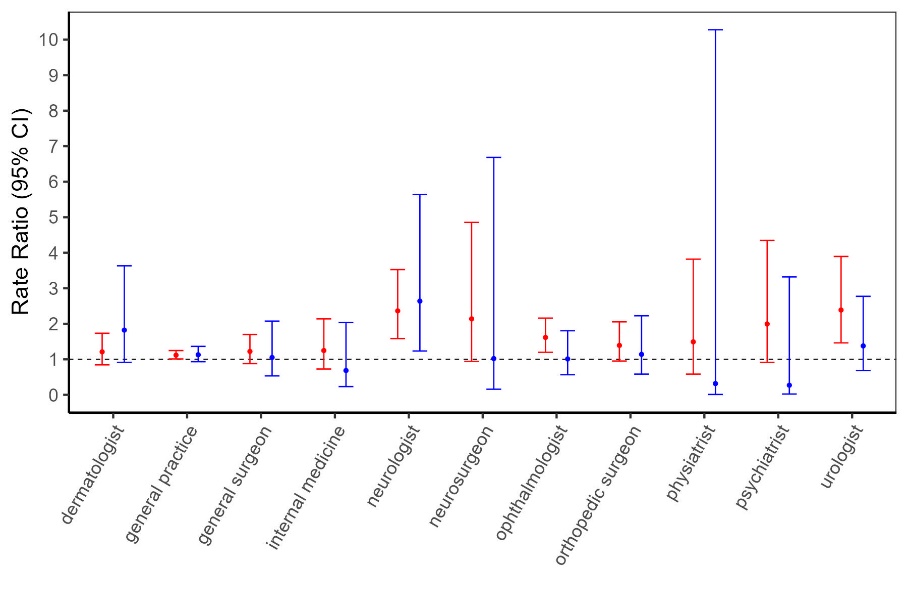


c) d)

**Supplemental Figure 1:** Multiple sclerosis (MS) cases versus controls in the five years before MS symptom onset: sex-specific rate ratios for (a) physician visits and (b) hospitalizations per International Classification of Diseases-9/10 chapter, (c) physician visits per specialty, and (d) prescriptions-filled per Anatomical Therapeutic Chemical (ATC) level 1 classification. Rate ratios were not statistically different between men and women.


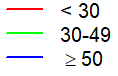

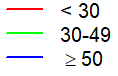

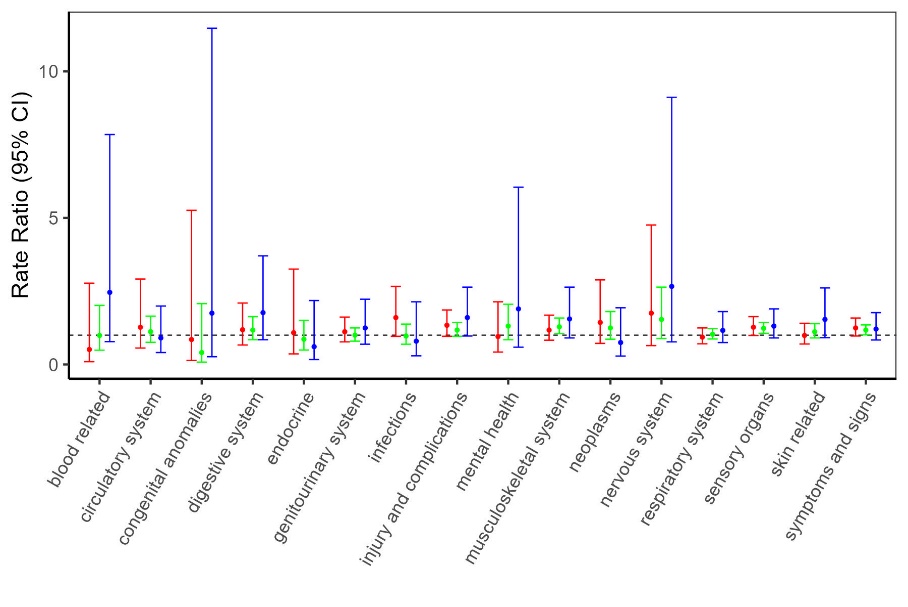

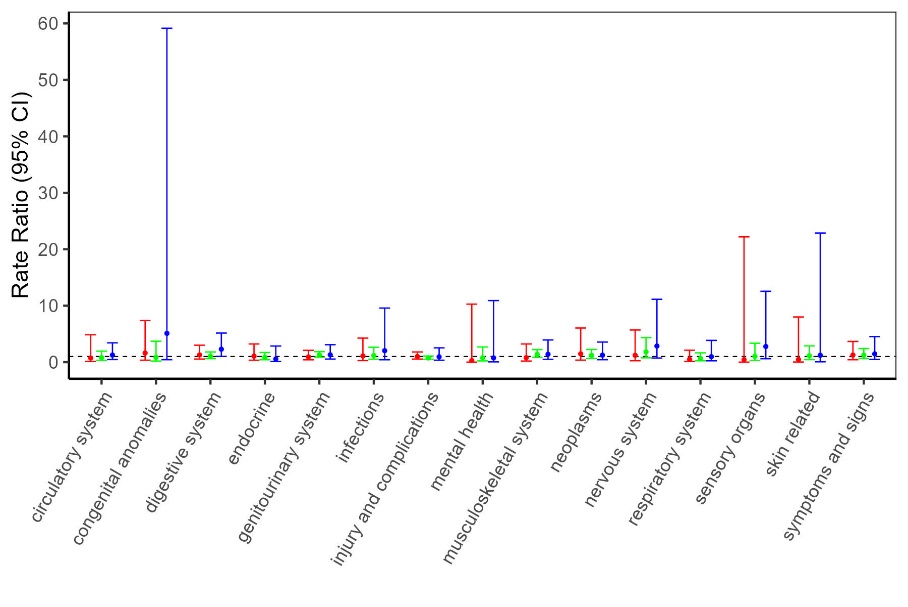
a) b)


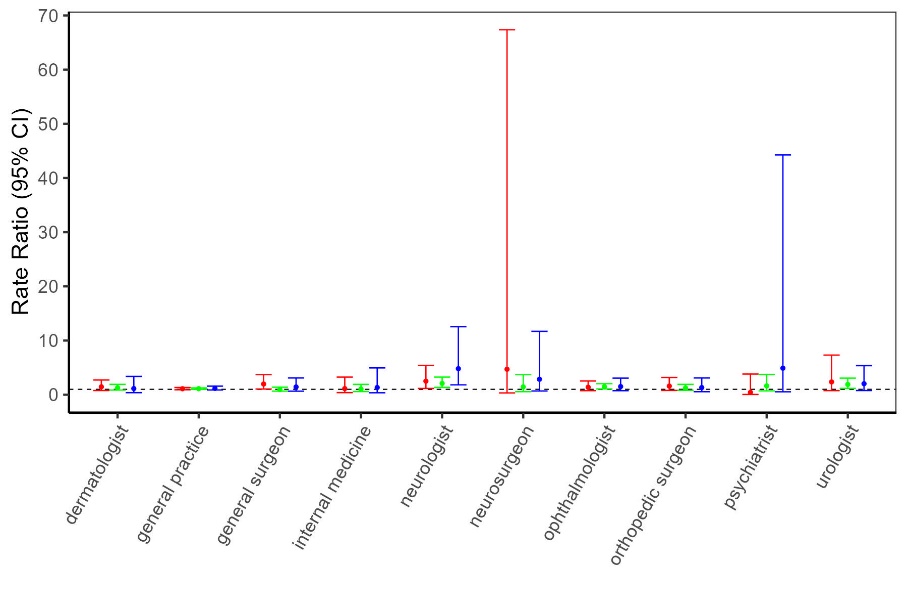

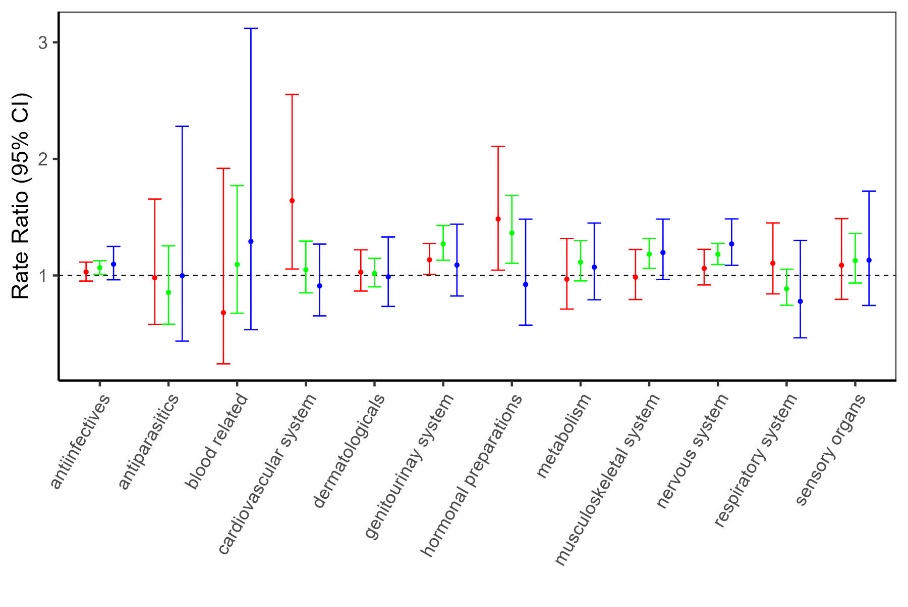


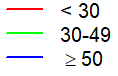

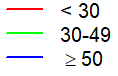
c) d)

**Supplemental Figure 2:** Multiple sclerosis (MS) cases versus controls in the five years before MS symptom onset: age-specific rate ratios for (a) physician visits and (b) hospitalizations per International Classification of Diseases-9/10 chapter, (c) physician visits per specialty, and (d) prescriptions-filled per Anatomical Therapeutic Chemical (ATC) level 1 classification. Rate ratios were not statistically different across age groups.


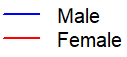

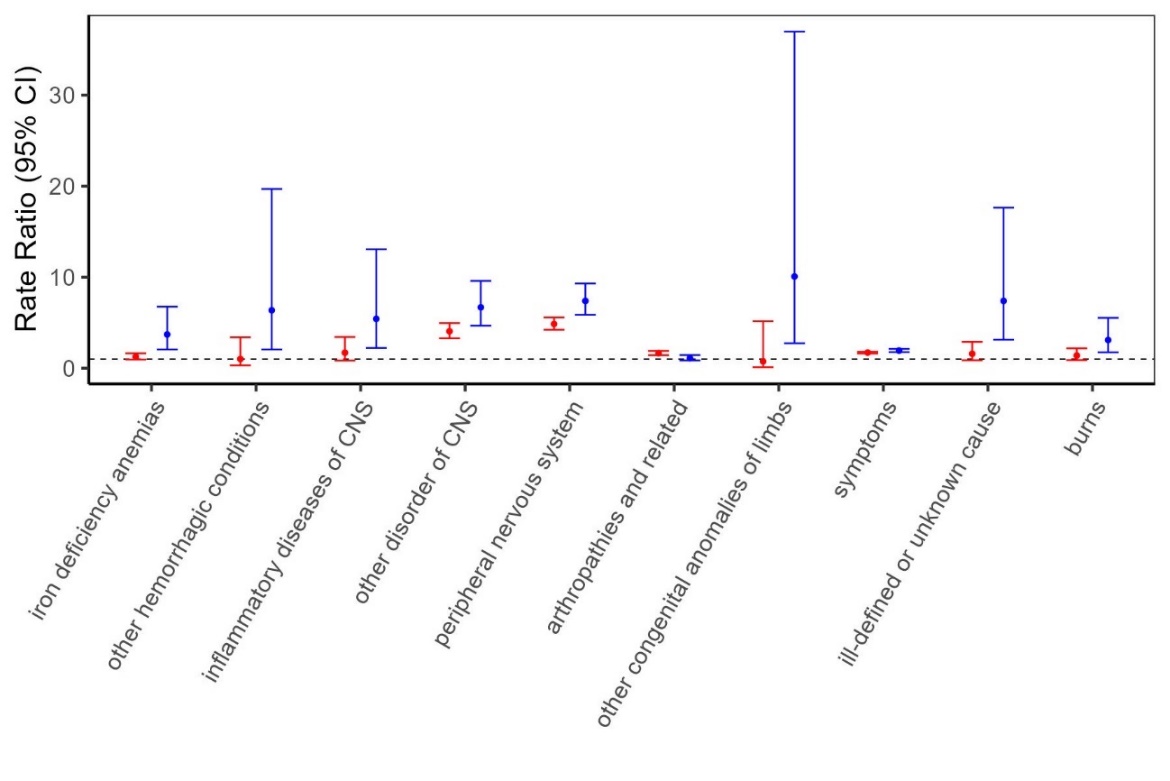


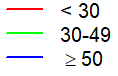

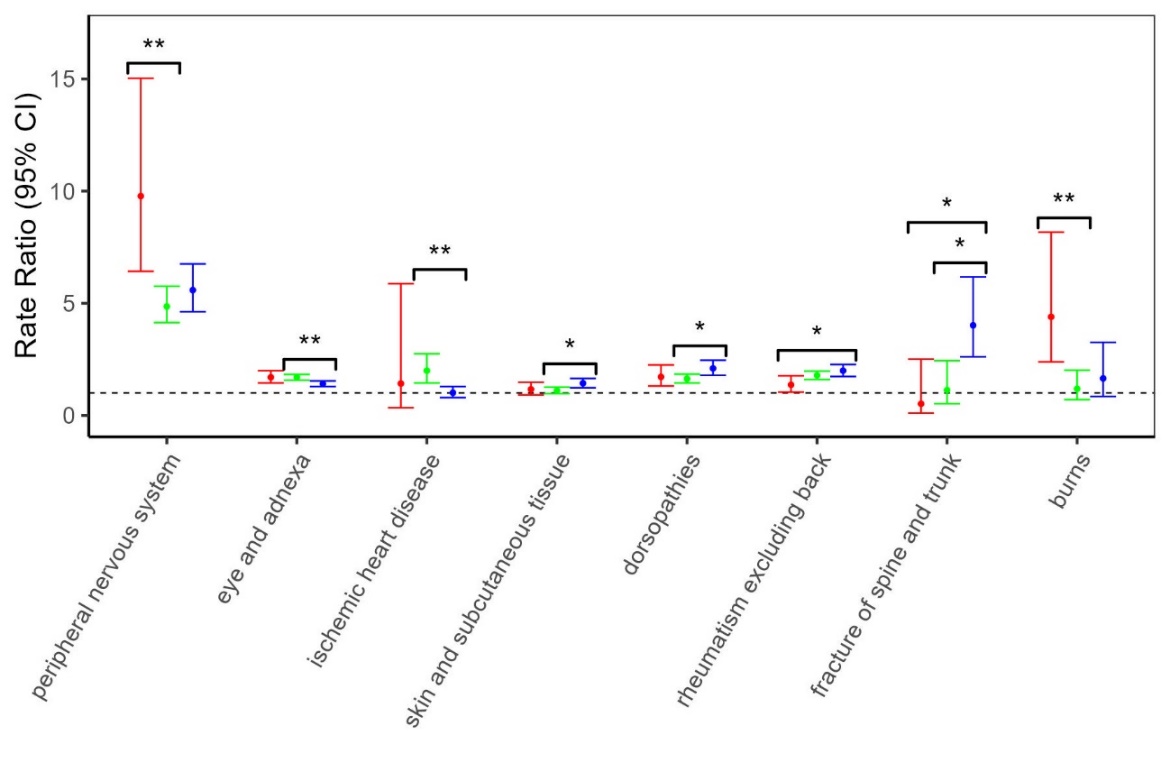


**Supplemental Figure 3:** Multiple sclerosis (MS) cases versus controls in the five years before the first demyelinating diagnostic code: sex- and age-specific rate ratios for physician visits per International Classification of Diseases-9 sub-chapter. Only sub-chapters that showed significant sex or age-based differences via likelihood ratio tests are displayed. * P < 0.05, ** P < 0.01, *** P < 0.001 indicates that the rate ratios are statistically different across age groups, based on Bonferroni tests.


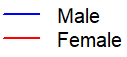

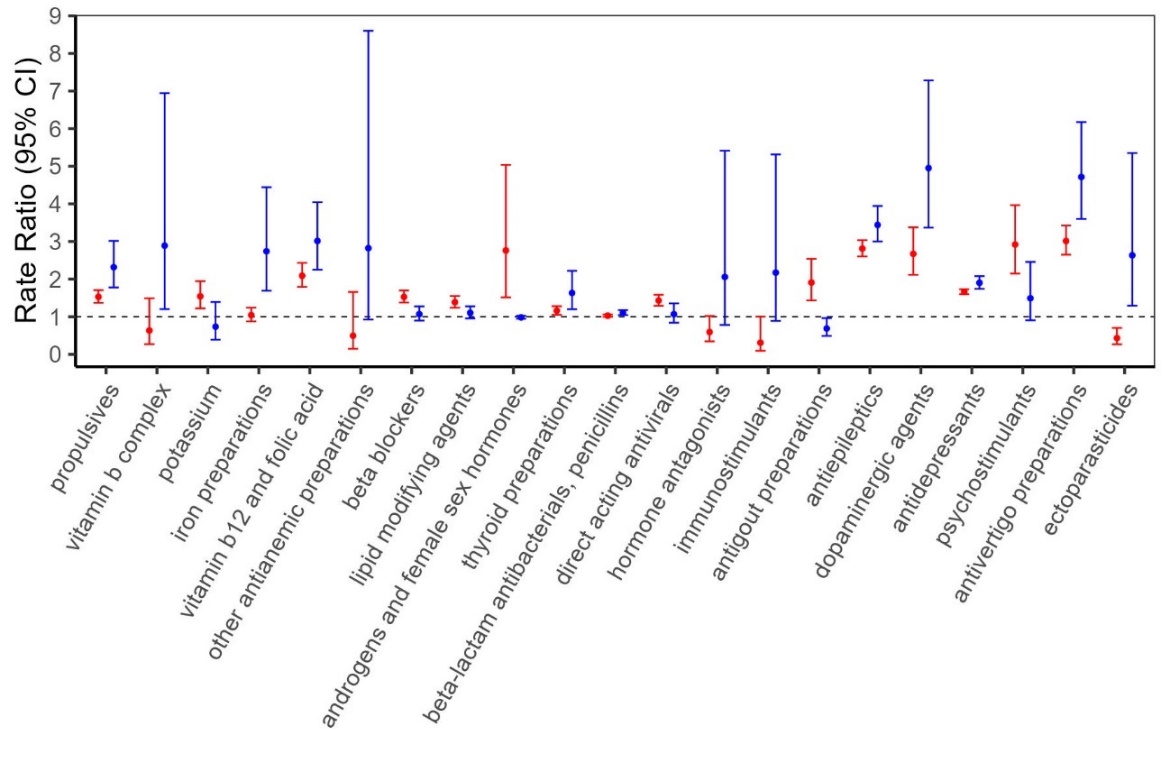


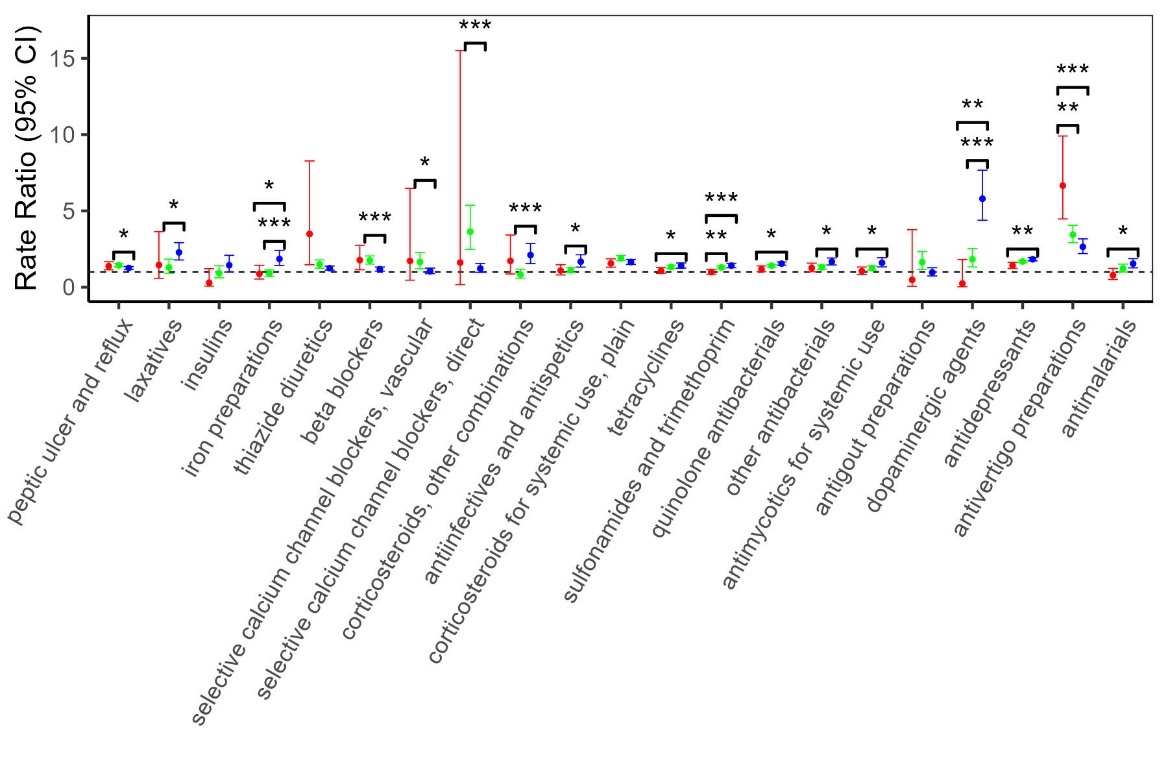

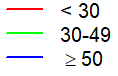


**Supplemental Figure 4:** Multiple sclerosis (MS) cases versus controls in the five years before the first demyelinating diagnostic code: sex- and age-specific rate ratios for prescriptions-filled per Anatomical Therapeutic Chemical (ATC) level 3 classification demyelinating claim. Only prescription classes that showed significant sex or age-based differences via likelihood ratio tests are displayed. * P < 0.05, ** P < 0.01, *** P < 0.001 indicates that the rate ratios are statistically different across age groups, based on Bonferroni tests.


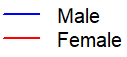

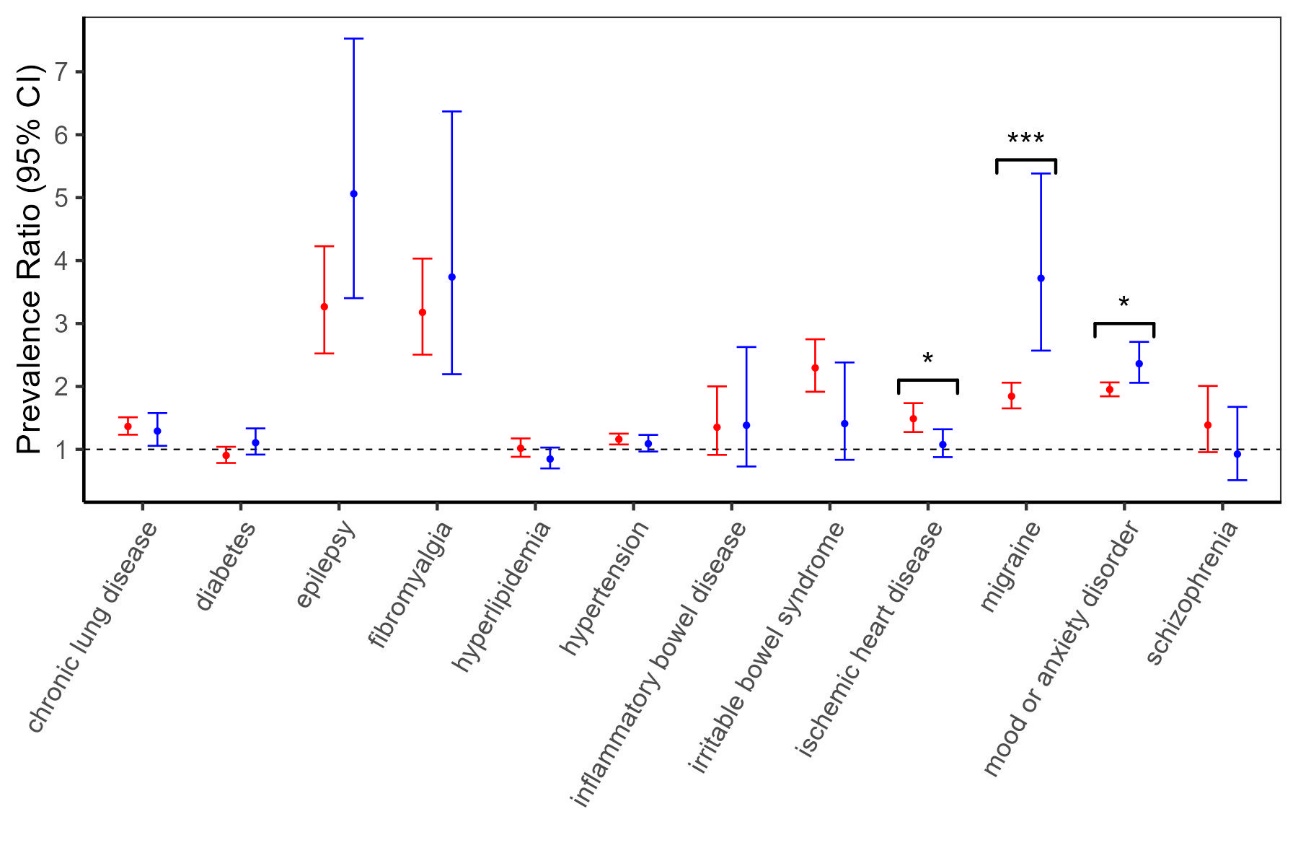


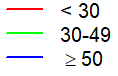

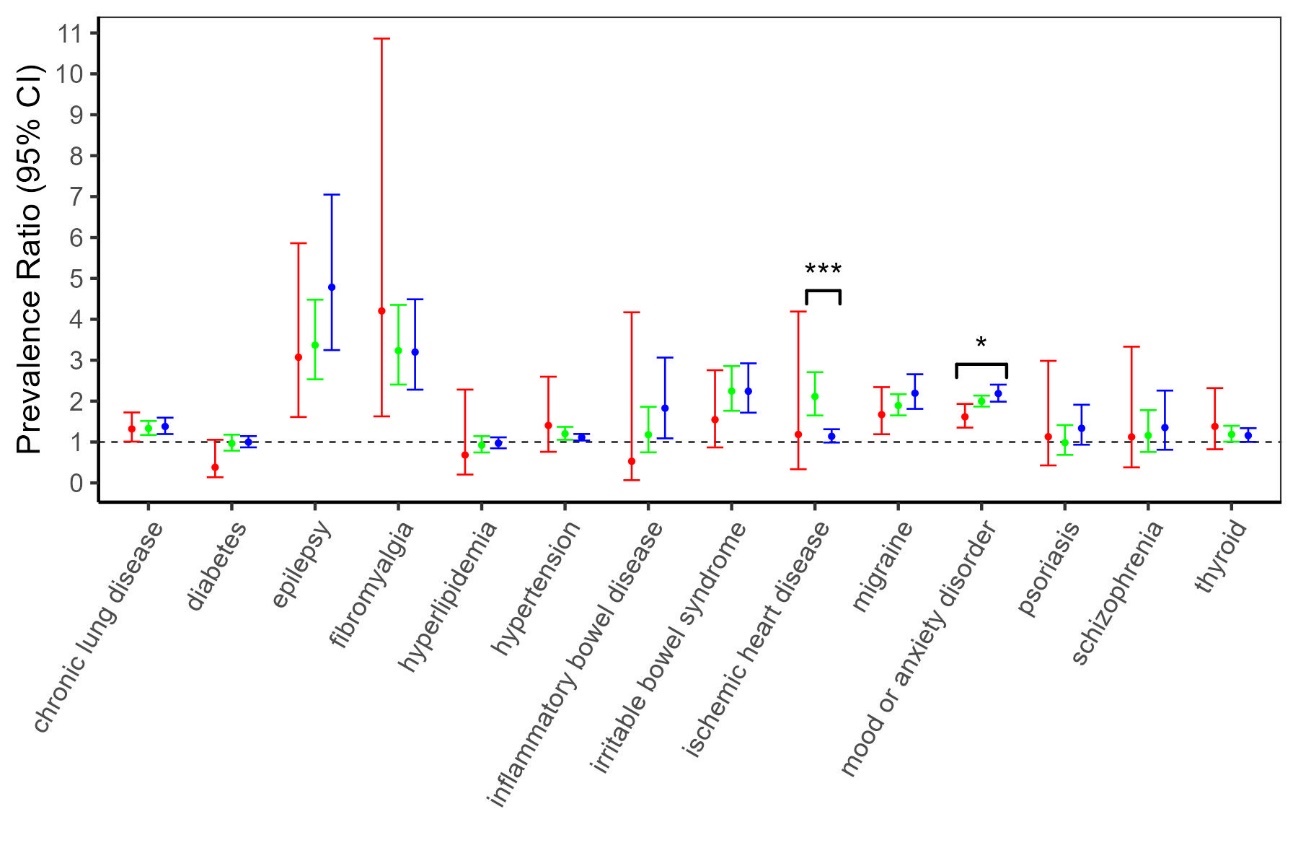


**Supplemental Figure 5:** Multiple sclerosis (MS) cases versus controls in the five years before the first demyelinating diagnostic code: sex- and age-specific prevalence ratios for the comorbidities. * P < 0.05, ** P < 0.01, *** P < 0.001 indicates that the rate ratios are statistically different between men and women or across age groups.
